# Sweet and Bright: Illuminating Glycoprotein-Mediated Endocytosis via Metabolic Labeling and NanoLuciferase

**DOI:** 10.1021/acschembio.6c00344

**Published:** 2026-06-07

**Authors:** Mai O. Soliman, Artturi Koivuniemi, Vincent Freiburghaus, Stefania Garbujo, Laurin Urech, Gianni Frascotti, Davide Prosperi, Miriam Colombo, Martina Hanzlikova, Nina Hartrampf, Timo Laaksonen, Shiqi Wang

**Affiliations:** † Division of Pharmaceutical Chemistry and Technology, Faculty of Pharmacy, 124454 University of Helsinki, Helsinki 00014 Finland; ‡ Institute of Biotechnology, Helsinki Institute of Life Sciences, University of Helsinki, Helsinki 00014, Finland; § Department of Pharmaceutics, Faculty of Pharmacy, Alexandria University, Alexandria 5372066, Egypt; ∥ Division of Pharmaceutical Biosciences, Faculty of Pharmacy, 3835 University of Helsinki, Helsinki 00014, Finland; ⊥ Department of Chemistry, 27217 University of Zurich, Winterthurerstrasse 190, Zurich 8057, Switzerland; # Department of Biotechnology and Biosciences, 9305 University of Milano-Bicocca, Piazza della Scienza 2, Milan 20126, Italy; ∇ Faculty of Engineering and Natural Sciences, Tampere University, Tampere 33014, Finland

## Abstract

Glycoprotein-mediated endocytosis is a critical pathway for the cell entry of biomolecules, pathogens, and delivery vectors. Despite this, glycans are among the most analytically challenging biomolecules due to their structural complexity and dynamic behavior. Here, we report a sensitive, membrane-specific, mix-and-read bioluminescent assay to monitor cell surface glycan dynamics during endocytosis. By combining metabolic labeling and bioorthogonal chemistry with split nanoluciferase, a bright luminescent enzyme comprising two complementary peptides, HiBiT and LgBiT, we were able to differentiate between the glycan uptake of four distinct cell-penetrating peptides (CPPs) reported to have varying degrees of glycan engagement. This proof-of-concept approach provides a basis for a broader understanding of glycan dynamics during endocytosis and may support the discovery of new ligands that exploit this pathway for cellular entry.

## Graphical abstract

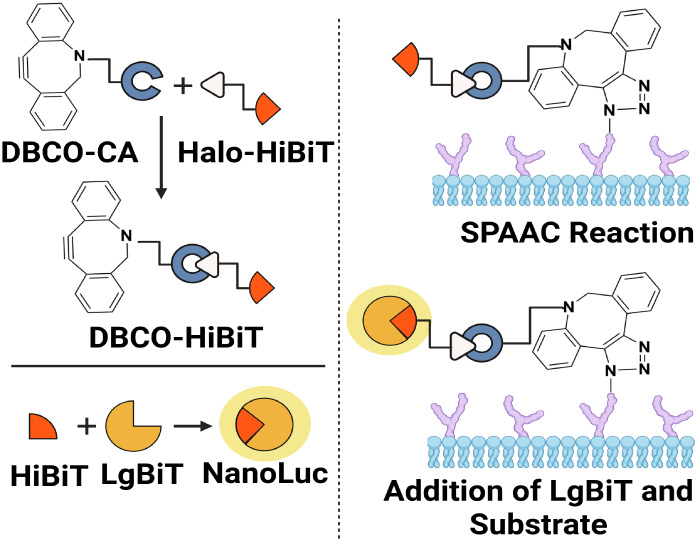

## 1 Introduction

The glycocalyx is a complex layer coating the outer surface of the cell membrane. It is composed of glycoproteins and proteoglycans, forming a dynamic boundary between the cell and its surroundings.^1,2^ Previously considered a mere passive barrier to molecular entry, it is now understood to be a far more functionally complex regulator of uptake.^3–7^ Several glycoproteins within the glycocalyx are now recognized as canonical receptors facilitating cellular ligand internalization,^6,8^ while also acting as molecular sieves for anionic macromolecules.^9^ It is therefore unsurprising that glycan-mediated uptake is implicated in cellular processes relevant to various disease states.^10,11^ These findings underscore their importance in revealing therapeutic targets and guiding delivery design.

However, studying glycoprotein engagement in endocytosis is inherently difficult. This is because glycosylation in live cells is a dynamic, non-template-driven process.^12–14^ While fluorescence imaging combined with metabolic labeling (incorporating functional groups in glycans) and bioorthogonal chemistry (clicking the labeled glycans with fluorescent tags) has emerged as an elegant tool to overcome this challenge,^15–18^ it carries innate drawbacks. Fluorophores may produce intracellular signals due to ongoing glycan biosynthesis and trafficking.^19–22^ Moreover, autofluorescence or light scattering may hinder the detection of subtle changes in cell surface glycan density during internalization.^23,24^ Additionally, real-time monitoring is limited by low throughput.^25,26^ Consequently, a method to directly assess surface glycan abundance in the native cellular context would be very valuable.

Bioluminescent assays have emerged as an alternative platform to overcome challenges in fluorescence-based assays.^27^ In particular, the split nanoluciferase platform offers a notable advantage: the HiBiT peptide generates luminescence exclusively upon binding to its complementary fragment, LgBiT, which is inherently membrane-impermeable. Consequently, luminescence can originate only from HiBiT that remains accessible at the cell surface.^28,29^ This platform has been used by Boursier et al.^23^ to monitor G protein–coupled receptor internalization in live cells using genetically expressed HiBiT fusions. More recently, it has been used to probe various other cell surface receptors through genetic engineering.^30,31^

Inspired by this, we proposed that combining split nanoluciferase with metabolic labeling and bioorthogonal chemistry would enable exclusive detection of cell-surface glycans and thus enable precise analysis of glycan-dependent internalization in live cells (Figure 1). In this regard, we metabolically labeled surface glycans using GalNAz (N-azidoacetylgalactosamine tetraacylated), followed by chemical conjugation to HiBiT using a heterobifunctional linker, DBCO-CA, that clicks with both commercially available Halo-HiBiT protein through its chloroalkane side chain and to azides through the alkyne handle. Upon the addition of LgBiT and substrate, the DBCO-HiBiT-conjugated glycans generate bright luminescence.^28^ However, if glycan-mediated endocytosis occurs, the signal decreases relative to the amount of internalized DBCO-HiBiT. Since split nanoluciferase is highly sensitive, it allows detection down to 10^–19^ mol, mirroring low glycan levels of 10^–18^-10^–15^ mol/cell.^32^ Additionally, it enables high-throughput signal detection by a plate reader. In light of studying cargo, we termed this DBCO-HiBiT-LgBiT assay the DHL assay.

**Figure 1:**
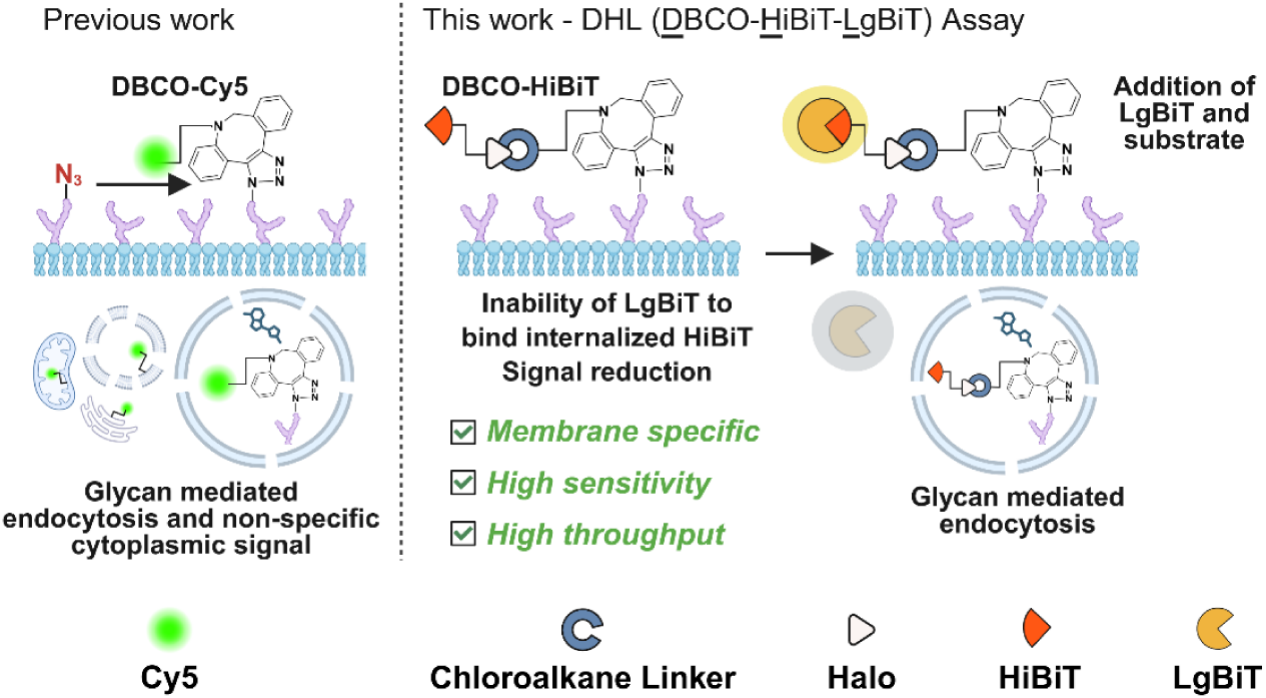
Schematic representation of the DHL assay. Left: Classic metabolic labeling and fluorescence-based click chemistry. Here, part of the signal could come from intracellular sources. Right: DHL assay, DBCO-CA heterobifunctional linker clicks with Halo-HiBiT using HaloTag technology. When added to cells, DBCO clicks with metabolically labeled azido glycans using SPAAC. This leaves HiBiT exposed, allowing the addition of complementary LgBiT and substrate to produce luminescence. Since LgBiT is membrane-impermeable, it is unable to access endocytosed HiBiT. The assay is more sensitive and enables high throughput. Created by Biorender.com.

## 2 Results and Discussion

### 2.1 DBCO-HiBiT Complex Synthesis and Characterizations

To establish the DHL assay, we first synthesized DBCO-HiBiT (Figure 2A). We incubated the DBCO-CA linker with commercially available Halo-HiBiT protein, leveraging the dehalogenase activity of Halo.^33^ Subsequently, we investigated the reaction efficiency by mass spectrometry (Figure 2B–C). Theoretically, the desired DBCO-HiBiT conjugate should give a mass shift of (+474 Da), which was indeed observed as the main peak with minimal trace peaks, indicating almost complete conversion within 1 h. To ensure that the Halo-HiBiT structure was not compromised postreaction, we performed a Western blot of the native protein and the protein postconjugation using an anti-HiBiT antibody. Both Halo-HiBiT and DBCO-HiBiT bands showed no significant difference, indicating maintenance of structural integrity (Figure 2D). To verify that Halo-HiBiT also retained its ability to complement LgBiT and generate luminescence, we compared the calibration curves of native Halo-HiBiT and postreaction.^34^ DBCO-HiBiT showed comparable luminescence to native Halo-HiBiT (Figure 2E). However, longer reaction time led to significant luminescent signal decay (Figure S1), which may stem from side reactions or conformational effects.^35^ Fresh Halo-HiBiT, used immediately without incubation in buffer, served as a reference for the luminescence output of nonconjugated HiBiT under optimal conditions.

**Figure 2:**
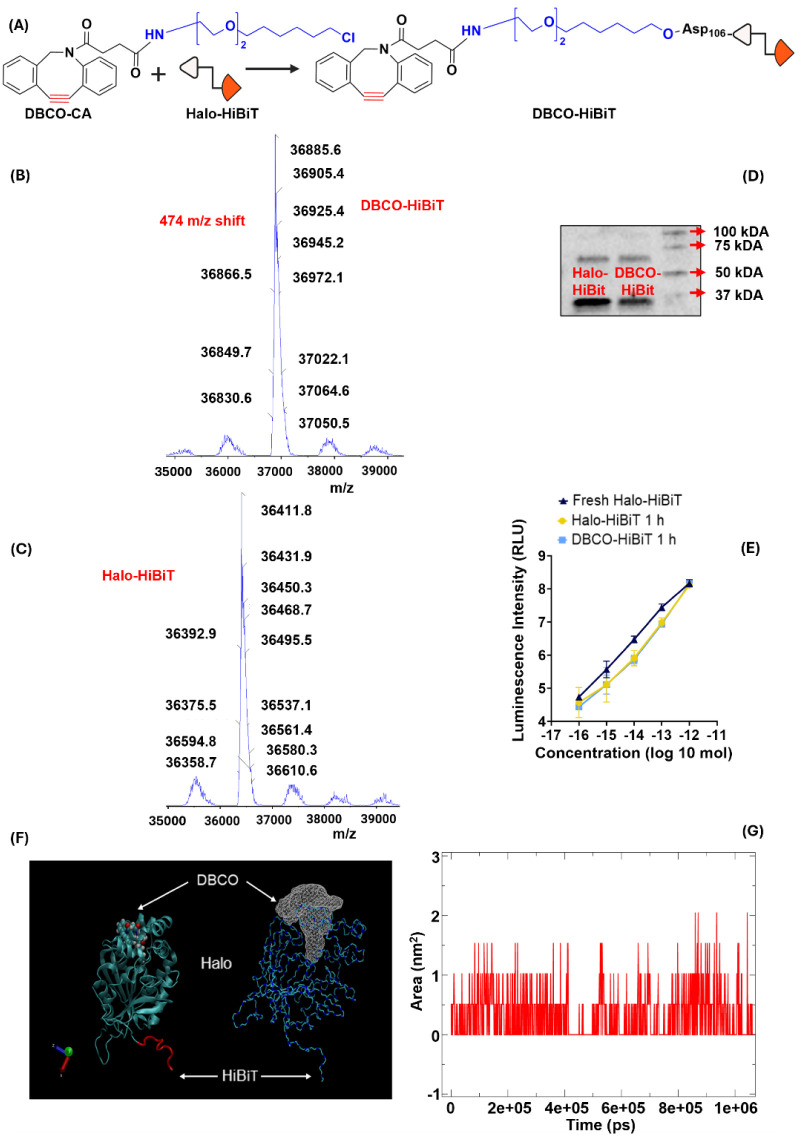
Characterizing DBCO-HiBiT conjugation. A) Schematic representation of DBCO-HiBiT conjugation. B) Mass spectra after conjugating Halo-HiBiT with DBCO-CA showing a mass shift of +474 Da. C) Halo-HiBiT mass spectra. D) Western blot of Halo-HiBiT and DBCO-HiBiT showing similar intensity bands. E) Calibration curve of fresh Halo-HiBiT (no incubation), Halo-HiBiT, and DBCO-HiBiT incubated in HBSS buffer for 1 h. F) Structural representation of Halo (cyan) fused to HiBiT (red) and conjugated with DBCO (left) and the spatial density map calculated from one simulation replicate (right). G) Solvent-accessible surface area of DBCO-CA as a function of simulation time.

We then investigated the availability of the DBCO alkyne handle in Halo-HiBiT postreaction, as it is crucial for the subsequent strain-promoted azide–alkyne cycloaddition (SPAAC) reaction. We carried out atomistic molecular dynamics (MD) simulation of DBCO-HiBiT in an aqueous environment and analyzed the solvent exposure of the alkyne group. The protein structure remained stable during simulations, with spatial density distribution and solvent-accessible surface area analysis revealing distinct fluctuations in the surface exposure of DBCO over time (Figure 2F–G). The alkyne handle displayed clear solvent accessibility in all replicates, suggesting its availability for further reactions.

### 2.2 Validation of SPAAC Reaction Using BRET

Following MD simulations, we sought to verify that the DBCO alkyne handle remained accessible in the DBCO-HiBiT complex for SPAAC cycloaddition. To this end, we used proximity-based BRET (bioluminescent resonance energy transfer) technique (Figure 3A).^36^ This technique relies on the distance-dependent energy transfer from a bioluminescent emitting donor to a fluorescent acceptor. In this case, the donor is nanoluciferase (after HiBiT and LgBiT complementation, emission peak at 460 nm), and the acceptor is a clickable azide-functionalized fluorophore (6-ROX, emission peak at 591 nm).^37^ It is expected that the reaction of DBCO-HiBiT with 6-ROX brings the donor and acceptor into close proximity, thereby producing a BRET signal, which is observed as an increase in the acceptor emission in the luminescence measurement. We used a no-alkyne control composed of Halo-HiBiT and 6-ROX to demonstrate that the BRET signal was specific to the DBCO-mediated SPAAC reaction rather than the nonspecific association of 6-ROX with Halo-HiBiT. We also included Halo-HiBiT without 6-ROX as a no-ligand negative control for baseline comparison.

**Figure 3:**
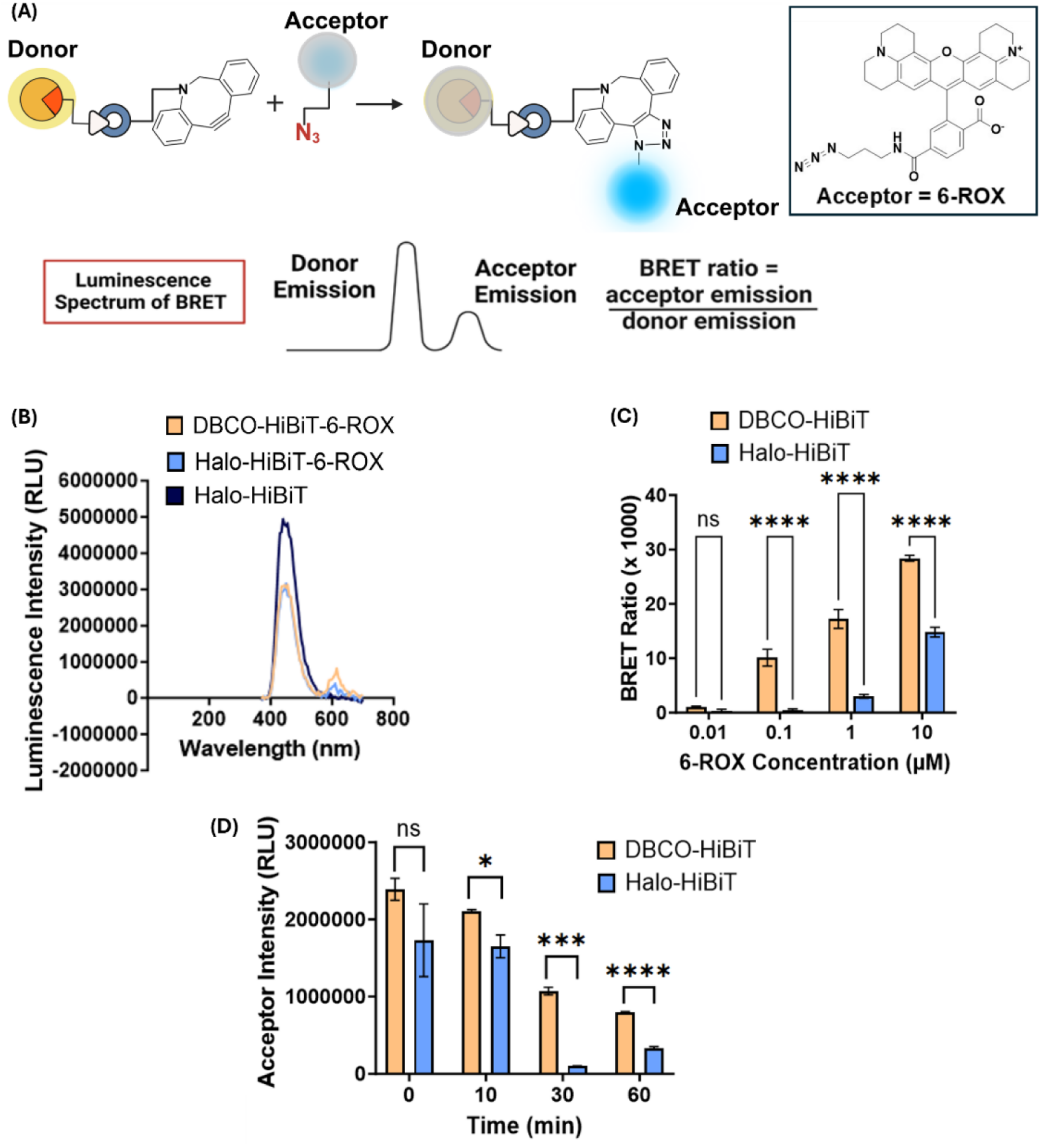
BRET assay. A) Schematic representation of the BRET assay. B) Luminescence spectra of DBCO-HiBiT with 6-ROX (SPAAC reaction), Halo-HiBiT with 6-ROX (no-alkyne control), and Halo-HiBiT without 6-ROX (no-ligand control). C) Evaluation of the BRET ratio of DBCO-HiBiT vs Halo-HiBiT (no-alkyne control) in relation to different concentrations (0.01–10 μM) of 6-ROX. D) Time-dependent acceptor bioluminescence intensity in the BRET assay at 0–60 min intervals. The concentration of the BRET donor, DBCO-HiBiT or Halo-HiBiT, was 5 nM for all samples. Statistical analysis was performed by two-way ANOVA. ****p < 0.0001, ***p < 0.001, **p < 0.01, *p ≤ 0.05, (ns) not significant: *p* > 0.05. Data is expressed as mean ± SD (*n* = 3).

As shown in the luminescence spectra (Figure 3B), in the no-ligand control, we observed only the donor nanoluciferase bioluminescence at 460 nm. In contrast, we observed the acceptor emission peak for 6-ROX after the SPAAC reaction with DBCO-HiBiT, which was nearly twice as great as in Halo-HiBiT (no-alkyne control). In order to further investigate how fast and how efficiently the SPAAC reaction proceeds at low reactant concentrations, we compared BRET ratios (acceptor emission/donor emission) at different 6-ROX concentrations (0.01–10 μM). The BRET ratio of DBCO-HiBiT was significantly higher than the no-alkyne control for the majority of tested concentrations (Figure 3C), indicating a successful and sensitive SPAAC reaction between DBCO-HiBiT and 6-ROX even at submicromolar concentrations. We next performed a time-course BRET experiment to evaluate the reaction kinetics between DBCO-HiBiT and 6-ROX compared to the no-alkyne control (Figure 3D). At zero time, no significant difference was observed between the two conditions. By 10 min, DBCO-HiBiT showed a statistically significant 1.27-fold increase in luminescence relative to the no-alkyne control. From 30 min onward, the luminescence of DBCO-HiBiT was approximately twice that of the control. These findings are consistent with previous reports of the inherently slow kinetics of SPAAC reaction.^38^

### 2.3 In Vitro Evaluation of Glycan Labeling by DHL Assay

After establishing alkyne accessibility, we set out to evaluate our DHL assay in live cells. We chose 4T1 as a model cell line for its robust incorporation of azido sugars and successful metabolic labeling, as in previous works.^22,39,40^ We added GalNAz as the azido sugar, and its cytotoxicity was evaluated (Figure S2) to determine the optimal concentration with minimal toxicity.^41^ We then employed DBCO-Cy5 to confirm that 4T1 cells were successfully metabolically labeled and could be conjugated via SPAAC.^42,43^ As shown in Figure 4A, at 500 nM, DBCO-Cy5 showed a clear signal difference between the metabolically labeled cells (with GalNAz) and the unlabeled control (without GalNAz). However, at low nanomolar concentrations (5 and 50 nM), the fluorescent signal of Cy5 decreased significantly and approached background levels, indicating the detection limit of the fluorescence-based SPAAC labeling and flow cytometry readout.

**Figure 4:**
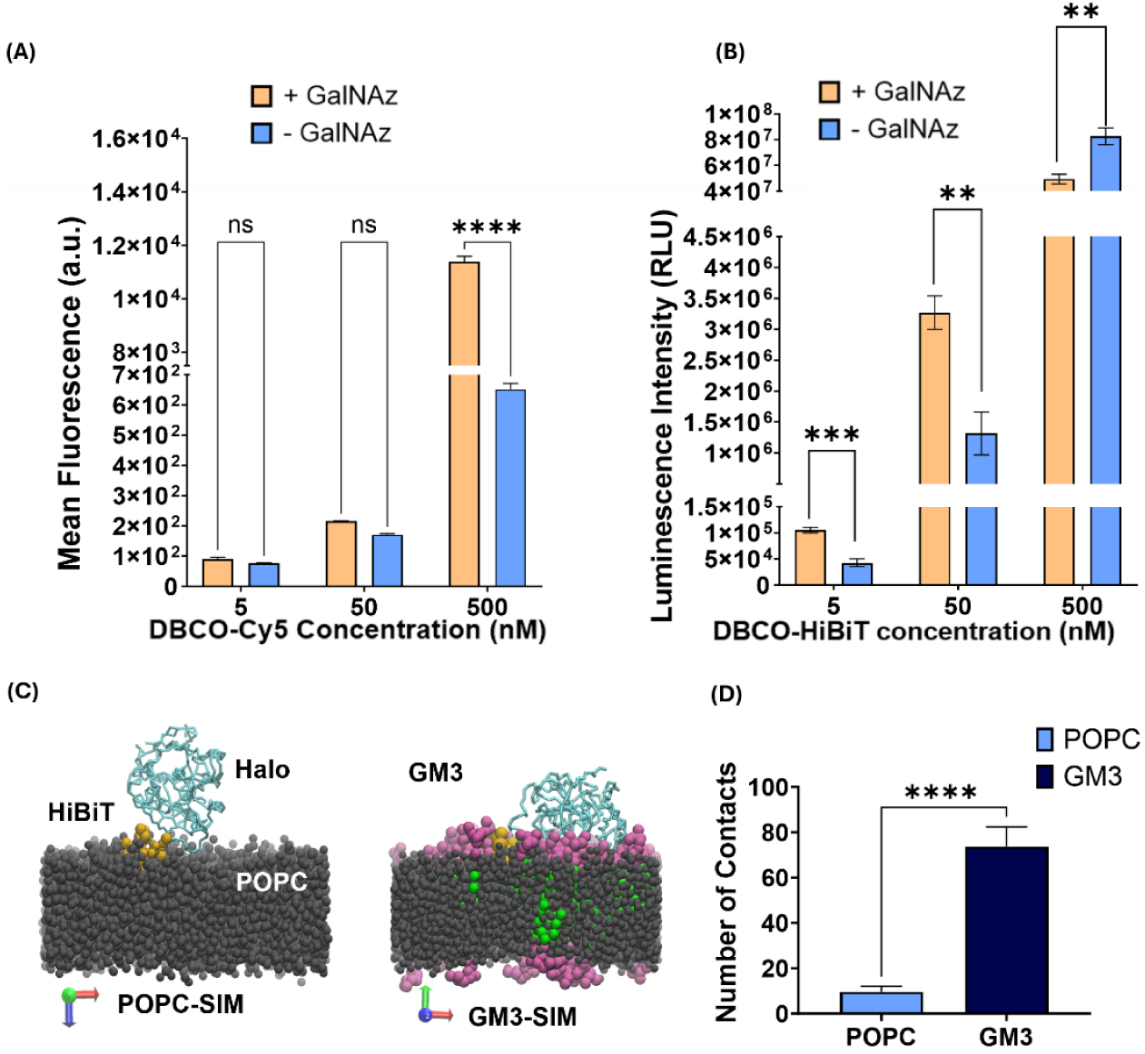
DHL assay-cell interactions. A) DBCO-Cy5 (5–500 nM) in 4T1 with or without GalNAz. B) DHL assay in 4T1 (5–500 nM) with or without GalNAz. C) Representative snapshots from molecular dynamics simulations illustrating Halo interactions with a POPC bilayer (left) and a GM3-enriched bilayer (right). POPC molecules are rendered as gray spheres. GM3 molecules are highlighted in green and pink spheres to emphasize lipid clustering around Halo (cyan). HiBiT is rendered as orange spheres. D) Quantification of the average number of contacts between Halo and POPC or GM3 lipids. Statistical analysis was performed by an unpaired Student’s *t*-test. ****p < 0.0001.

Then we labeled DBCO-HiBiT on 4T1 cells via SPAAC and compared our DHL assay to DBCO-Cy5. As shown in Figure 4B, our DHL assay revealed significantly increased signal in metabolically labeled cells relative to the nonmetabolically labeled control at 5 and 50 nM. However, we also observed an increase in background at 500 nM, indicating possible membrane saturation. We hypothesized that this high background signal likely reflected the high sensitivity of nanoluciferase and possible nonspecific binding between DBCO-HiBiT and the cell membrane at such relatively high concentrations. To test this hypothesis, we replaced the nanoluciferase by a control protein, DBCO-GFP, using the same conjugation strategy via Halo-GFP^44^ and DBCO-CA linker. Then we investigated the labeling of DBCO-GFP on 4T1 with or without GalNAz using flow cytometry at 500 nM (Figure S3). GalNAz-labeled 4T1 showed a significantly higher signal, while the background signal was comparable to that of the negative control (without DBCO-GFP). This suggests the increase in background signal of our DHL assay at 500 nM is not due to the SPAAC reaction itself (lack of selectivity or low efficiency), but the nanoluciferase.

To investigate potential reasons for the increased background signal observed at high DBCO-HiBiT concentrations, we carried out coarse-grained MD simulations and quantified contacts between the protein (Halo part) and phosphatidylcholine (POPC) lipid membranes with or without GM3 gangliosides (model glycosphingolipid). We hypothesized that, even in the absence of GalNAz labeling, strong interactions between Halo-HiBiT and lipids could occur and contribute to the background signal by promoting the strong association of Halo-HiBiT with the cell membrane, especially at high DBCO-HiBiT concentrations. GM3 was chosen for the study as it is the most abundant negatively charged glycolipid in mammalian cell membranes and, therefore, is expected to represent a significant fraction of glycosphingolipids present in our experimental system.

Representative simulation snapshots show that in POPC-only bilayers, the Halo-HiBiT made minimal contacts with the surrounding lipids, although the amphiphilic HiBiT segment was often but transiently associated with lipids (Figure 4C). By contrast, in GM3-enriched bilayers, GM3 molecules clustered around the protein, forming extensive interactions with Halo-HiBiT. The average number of contacts with GM3 was markedly higher compared to POPC, demonstrating a clear preference for Halo-HiBit in such interactions (Figure 4D). A more detailed analysis revealed that GM3 lipids interact predominantly via electrostatic interactions with Halo-HiBiT (data not shown). Interestingly, the HiBiT segment, which contains only three positively charged residues (two lysines and one arginine), was consistently embedded within the GM3 headgroup region and formed stabilizing electrostatic interactions with these lipids. Together, these data suggest that the HiBiT segment may facilitate the strong association of Halo-HiBiT with GM3-rich membranes, potentially improving the interaction with GalNAz and increasing reaction specificity toward negatively charged glycolipids. However, this strong association may also contribute, at least in part, to the background signal observed in our experiments, particularly at higher probe concentrations. It is also important to note that membrane association of the HiBiT segment likely limits its accessibility to LgBiT, thereby impairing efficient complementation and luminescence generation.

### 2.4 In Vitro Evaluation of Glycan-Dependent Endocytosis by DHL Assay

Despite the nonspecific interactions between DBCO-HiBiT and cell membranes at higher concentrations, the DHL assay reliably detected labeled glycans above background at an optimal concentration of 5 nM. We used this condition to investigate whether the DHL assay could monitor cell-surface glycan abundance. We focused on glycosaminoglycans (GAGs) since they are major endocytosis-regulating components of the glycocalyx.^5,45,46^

We first confirmed that GAGs could be metabolically labeled by GalNAz. After GalNAz treatment, we enzymatically removed two representative GAGs, heparan sulfate (HS) or chondroitin sulfate (CS), using corresponding glycosidases.^47^ The HS and CS enzymatic removal protocol was optimized and confirmed by comparing treated cells with controls via immunofluorescence imaging (Figure S5). Then, we incubated heparinase- or chondroitinase-digested cells with DBCO-Cy5 or DBCO-HiBiT and characterized the labeling by flow cytometry or the DHL assay. In both cases (Figure 5A, 5B), the labeling did not significantly change upon removal of HS but significantly decreased upon removal of CS. This is in line with previous literature, where GalNAz is moderately incorporated into CS via O-linked α-GalNAc residues, but the azido group is cleaved during HS glycosylation.^48–50^ However, upon CS removal, the nonmetabolically labeled control (without GalNAz) showed significantly increased fluorescence in the DBCO-Cy5 assay, suggesting that enzymatic removal of CS resulted in possible DBCO-Cy5 aggregation. In contrast, DHL assay results demonstrated that the nonmetabolically labeled control remained the same upon CS or HS removal, indicating a more robust signal when glycan type and density are altered, which is needed in endocytosis studies.

**Figure 5:**
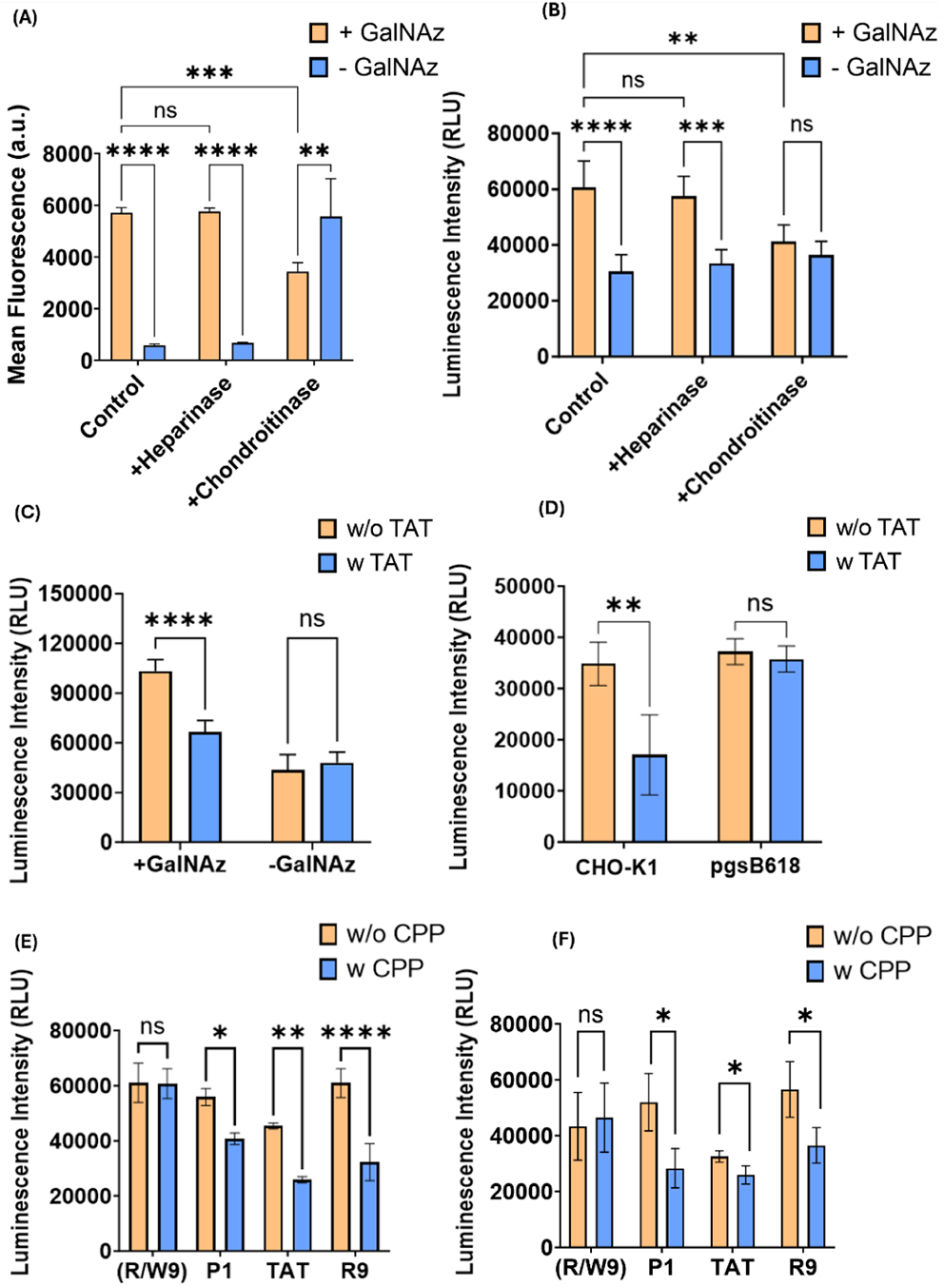
Investigating glycan engagement in CPPs internalization by DHL assay. A) 4T1 cells were metabolically labeled and subjected to heparinase or chondroitinase digestion to remove HS or CS. The corresponding GAGs labeling and removal were evaluated after conjugation to DBCO-Cy5. B) 4T1 cells were metabolically labeled and subjected to heparinase or chondroitinase digestion to remove HS or CS. The corresponding GAGs labeling and removal were evaluated by the DHL assay. C) DHL assay in 4T1, determining TAT-glycan-mediated endocytosis. Without GalNAz represents the nonmetabolically labeled control. D) TAT-glycan assay validation using metabolically labeled CHO-K1 and its mutant w/wo TAT. E) Four different CPPs: (R/W)­9, P1, TAT, and R9 were analyzed in metabolically labeled 4T1. Glycan interactions of the 4 CPPs were determined as a signal decrease after CPP addition w or w/o CPP. F) Glycan interactions of the 4 CPPs determined as signal decrease after CPP addition w or w/o CPP after the enzymatic removal of CS. Statistical analysis was performed by two-way ANOVA. ****: *p* < 0.0001, ***: *p* < 0.001, **: *p* < 0.01, *: *p* ≤ 0.05, (ns) not significant: *p* > 0.05. Data is expressed as mean ± SD (*n* = 3).

To test the DHL assay’s ability to quantify glycan-mediated endocytosis, as a model cargo, we used TAT, a CPP that is reported to rely on GAGs for endocytosis (Figure 5C).^51–53^ Upon TAT addition, the signal significantly decreased, corresponding to glycan-mediated endocytosis. The background signal (without GalNAz) remained approximately the same with or without TAT, suggesting assay specificity toward glycan endocytosis. We also performed the TAT uptake at 4 °C as a control condition to inhibit endocytosis.^54^ The DHL assay results at 4 °C showed no significant difference in signal after TAT addition, indicating that the signal decrease is associated with energy-dependent endocytosis (Figure S5). To further validate that the signal change is due to glycan-mediated endocytosis, we employed CHO-K1 cells (wild-type) and their mutant pgsB618 (GAG-deficient) (Figure 5D).^55^ Cytotoxicity of GalNAz was investigated to inform the optimal concentration with the least toxicity. After TAT addition, CHO-K1 demonstrated a significant signal decrease, in line with 4T1 cells and previous results.^53^ In contrast, the signal decrease was nonsignificant in pgsB618 due to GAG loss and impaired glycosylation.^56–60^ This is in line with the literature, where TAT internalization is GAG-dependent.

To further explore the applicability of the DHL assay in glycan-mediated internalization, we evaluated 4 CPPs in 4T1 cells, each reported to have a different glycan interaction profile. As can be seen in Figure 5E, R9 and TAT showed the most significant signal decrease, which is indeed in line with previous studies on their relatively more intricate reliance on GAGs for endocytosis. P1, on the other hand, is an amphiphilic peptide that resulted in a milder decrease in signal, indicating less dependence on glycan-mediated endocytosis for uptake. R/W9 is reported to be internalized independent of glycan-mediated interactions and, indeed, has shown virtually no difference in signal.^51,51,61^ We have also evaluated such CPP uptake in the absence of CS (Figure 5F), which interacts with most of the used CPPs but to a lesser extent than HS. The results showed no significant change in signal after the addition of R/W9, consistent with its independence from GAGs. The two peptides, TAT and R9, showed a modest but significant inhibition of signal, which correlates with previous literature showing that CS removal results in a modest decrease in these CPPs’ uptake.^62^ A similar decrease in the signal was also observed in P1, albeit to a slightly larger extent. These results highlight the DHL assay’s ability to distinguish between peptides with varying levels of glycan involvement during uptake. The ability to capture such differences in live cells provides direct experimental confirmation of glycan-dependent mechanisms as reported in the literature and underscores the assay’s potential as a practical and reliable approach for comparing internalization behaviors across different biomolecules.

## 3 Conclusion

The interpretation of our results should take into account some limitations. Metabolic labeling inherently modifies only a subset of surface glycans, depending on their accessibility and glycosylation pathways involved, so the signal reflects the labeled pool rather than the full range. Moreover, nonspecific membrane binding of Halo-HiBiT may partially dilute this pool and should therefore be considered when interpreting assay results. These limitations define the current scope of the platform while also highlighting opportunities for future optimization of the labeling strategy. Nevertheless, relative glycan-dependent uptake remains clearly distinguishable. We conclude that the DHL assay could probe surface glycan variations in live cells. Our results showed that CPPs internalization is accompanied by a decrease in surface-labeled glycan, with the extent of reduction differing among peptides. The assay’s sensitivity and ease of use offer opportunities for discovering new ligands that exploit this pathway for cellular entry.

## Supplementary Material



## Data Availability

The original data are available in the Zenodo repository, with the DOI: 10.5281/zenodo.17657965.

## Notes

Figures (e.g., viability assays, DBCO-GFP signal-to-background ratio, validation of removal of GAGs, etc.), and detailed materials and experimental procedures (PDF)
