# Supplementary material for: Sweet and Bright: Illuminating Glycoprotein-Mediated Endocytosis via Metabolic Labeling and NanoLuciferase
Source: ACS Chem Biol. 2026 Jun 7;21(6):1576–84. doi: 10.1021/acschembio.6c00344 (PMC13288459; doi:10.1021/acschembio.6c00344)
Supplement: Supplementary file 1 [file cb6c00344_si_001.pdf]

Supplementary information for

Sweet and Bright: Illuminating Glycoprotein-Mediated Endocytosis via Metabolic Labeling and NanoLuciferase

Authors

Mai O. Soliman[*,a,b,c] , *Dr. Artturi Koivuniemi*[d], *Vincent Freiburghaus*[e], *Dr. Stefania Garbujo*[f], *Laurin Urech*[e], *Dr. Gianni Frascotti*[f], *Prof. Dr. Davide Prosperi*[f], *Prof. Dr. Miriam Colombo*[f], *Dr. Martina Hanzlikova*[a], *Prof. Dr. Nina Hartrampf*[e], *Prof. Dr. Timo Laaksonen*[d,g], *Prof. Dr. Shiqi Wang*[*,a,b]

[a:] Division of Pharmaceutical Chemistry and Technology, Faculty of Pharmacy, University of Helsinki, 00014 Helsinki, Finland

[b:] Institute of Biotechnology, Helsinki Institute of Life Sciences, University of Helsinki, 00014 Helsinki, Finland

[c:] Department of Pharmaceutics, Faculty of Pharmacy, Alexandria University, 5372066 Alexandria, Egypt

[d:] Division of Pharmaceutical Biosciences, Faculty of Pharmacy, University of Helsinki, 00014 Helsinki, Finland

[e:] Department of Chemistry, University of Zurich, Winterthurerstrasse 190, 8057 Zurich, Switzerland

[f:] Department of Biotechnology and Biosciences, University of Milano-Bicocca, Piazza della Scienza 2, 20126 Milan, Italy

[g] Faculty of Engineering and Natural Sciences, Tampere University, 33014 Tampere, Finland

[*:] Corresponding authors

Table of Contents

1. Supplementary Figures (S1-S6) S3

2. Materials and Experimental Methods S8

2.1 Materials S8
2.2 Synthesis and Evaluation of DBCO-HiBiT Conjugation S8
2.2.1 ESI-Q-TOF-LC-MS/MS 8
2.2.2 Western Blot S9
2.2.3 Calibration Curve S9
2.2.4 Atomistic Molecular Dynamics Simulations S10
2.3 Evaluation of DBCO-CA accessibility using proximity-based BRET S10
2.4 Optimization of GalNaz Metabolic Labeling S11
2.4.1 Cell culture S11
2.4.2 Viability Assay S12
2.4.3 DBCO-Cy5 labeling and flow cytometry analysis S12
2.4.4 DHL assay labeling analysis S12
2.4.5 Evaluation of membrane non-specific binding using Halo-GFP S13
2.4.6 Coarse-Grained Molecular Dynamics Simulations S13
2.5 Optimization and validation of DHL assay S14
2.5.1 GalNaz labeling specificity evaluation S14
2.5.2 Evaluation and validation of DHL assay ability to determine endocytosis S15
2.6 Determining glycan-CPP interactions and immunostaining S15
2.6.1 Validation of glycan-mediated endocytosis using CPPs S15
2.7 Data analysis methods S16
2.7.1 Statistical analysis S16
2.7.2 Dynamic simulation analysis S16
2.8 Cell-penetrating peptide synthesis S16
2.8.1 Reagents and solvents S16
2.8.2 Automated flow-based peptide synthesis (AFPS) S17
2.8.3 TFA-mediated peptidyl-resin cleavage and global deprotection S18
2.8.4 Liquid Chromatography with High-Resolution Electrospray Ionization Mass Spectrometry (LC-HR-ESI-MS) S18
2.8.5 Semi-Preparative Reverse-Phase High Performance Liquid Chromatography (RP-HPLC) S19
2.8.6 Synthesis of H_2N-RQIKIWFQNRRMKWKK-$CONH_2$ S19
2.8.7 Synthesis of H_2N-RRRRRRRRR-$CONH_2$ S21
2.8.8 Synthesis of H_2N-RWWRRWRR-$CONH_2$ S22
2.9 Recombinant Halo-GFP production and purification S23
2.10 Figure S12: Uncropped Blot for figure 1 in the main text S24
References S25

1. Supplementary Figures (S1-S6)

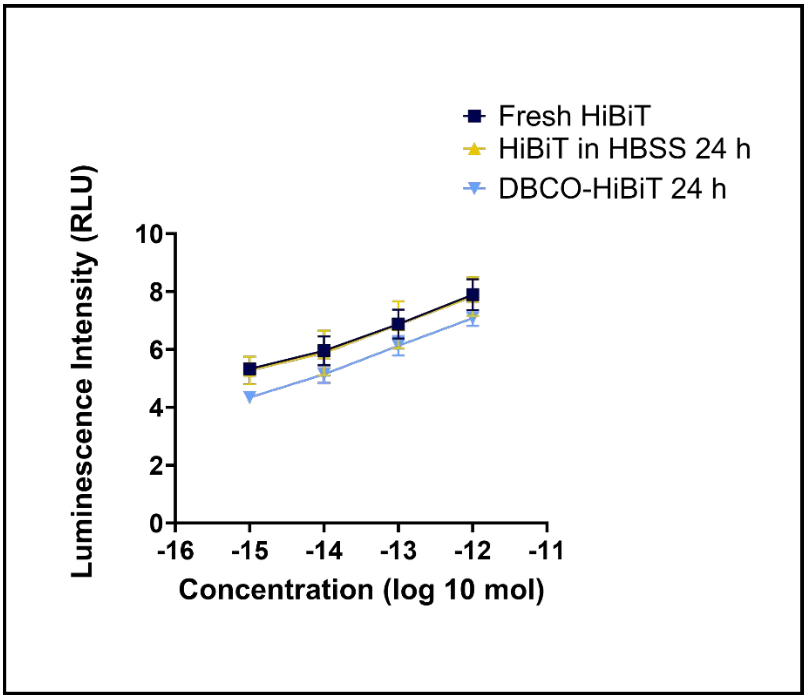

Figure S1: calibration curve of fresh Halo-HiBiT (5 nM) along with Halo-HiBiT (5 nM) and DBCO-HiBiT (5 nM 1:1 ratio) incubated in HBSS buffer for 24 h. Data is expressed as mean ± SD (n = 3).

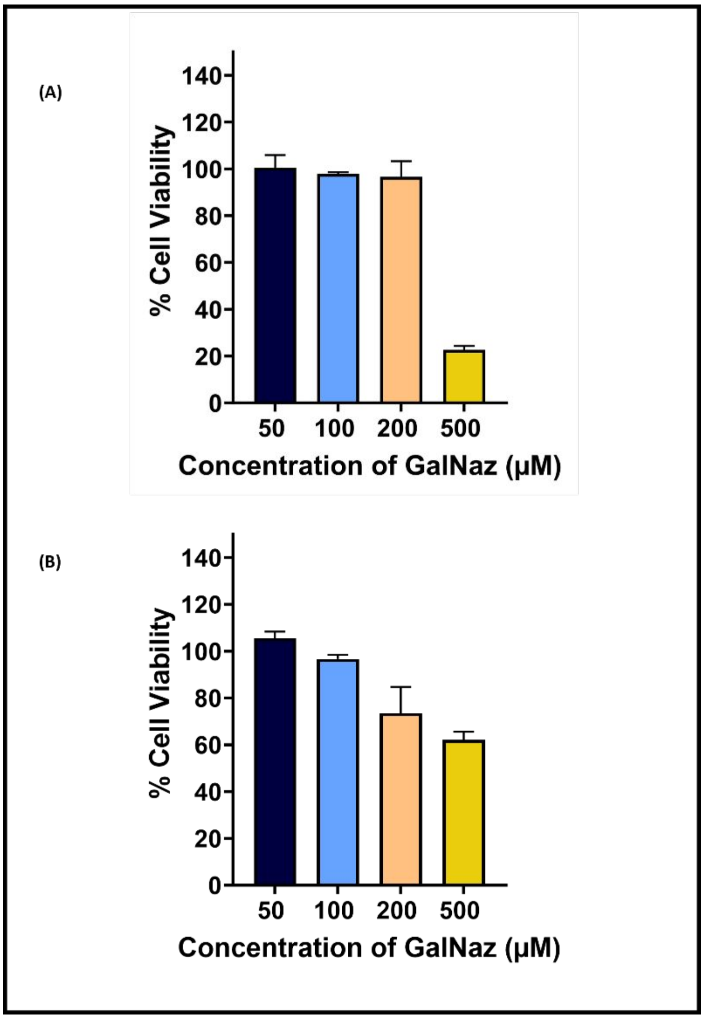

Figure S2: Cell viability assay (CellTiter-Glo®). A) 4T1 cells treated with increasing concentrations (50-500 μM) of GalNaz. **B)** CHO cells treated with increasing concentrations (50-500 μM) of GalNaz. Luminescence signals were normalized to the signal from untreated controls and reported as percentage cell viability. Data is expressed as mean ± SD (n = 3). The optimal concentration chosen was 100 μM for both cell lines.

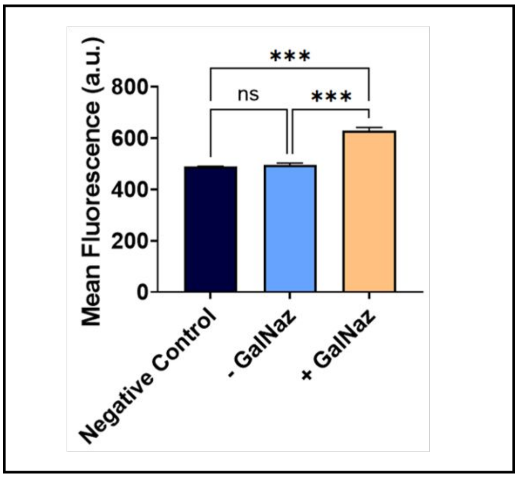

Figure S3: Validation of DHLassay membrane non-specific interactions by replacing Halo-HiBiT with Halo-GFP and using the same DBCO-CA linking strategy at 500 nM concentration (1:1 ratio) assayed using flow cytometry. Mean fluorescence intensity w or w/o GalNaz was compared vs negative GFP control. Statistical analysis was performed by one-way ANOVA. ***: p<0.001, (ns) not significant: p>0.05. Data is expressed as mean ± SD (n = 3).

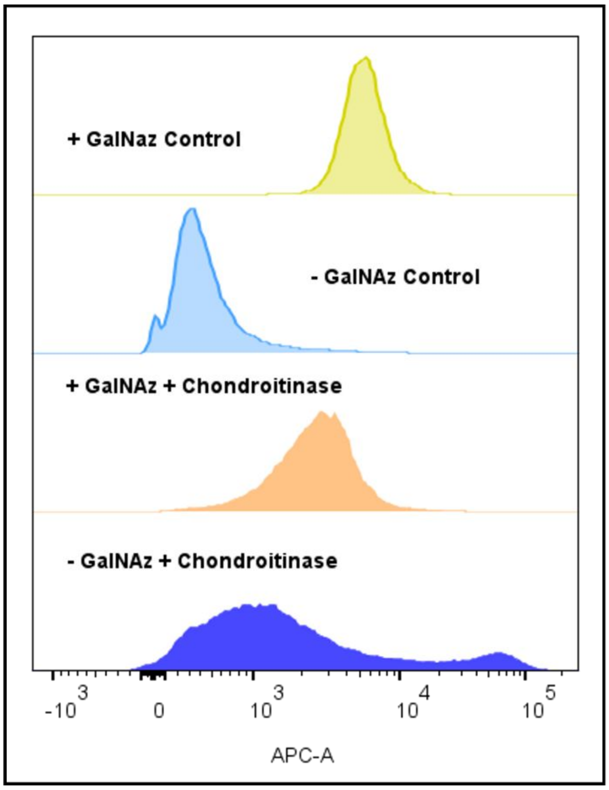

Figure S4: Flow cytometry results of 500 nM DBCO-Cy5 in the presence and absence of CS. Data analyzed by FlowJo v10.

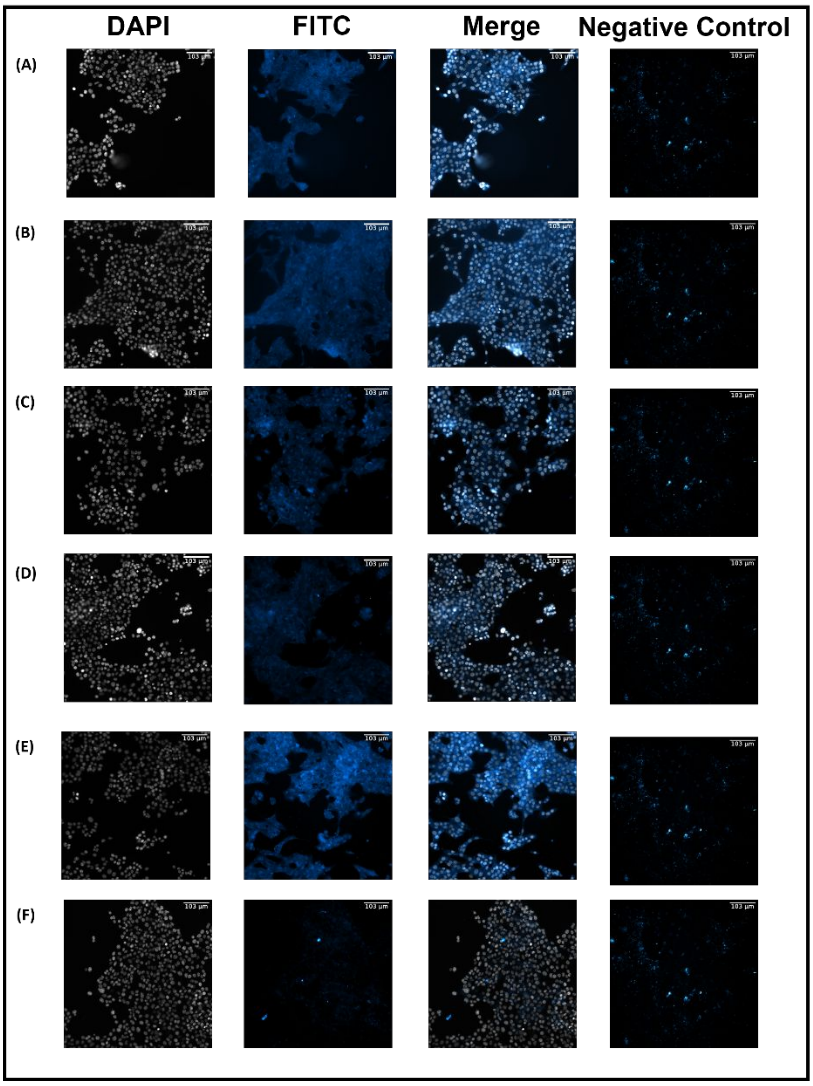

Figure S5: **Confocal microscopy images with DAPI/FITC dual staining in 4T1 cells with Chondroitinase ABC or Heparinase I and III. A)** Confocal images of positive control with Chondroitin Sulphate. **B)** Confocal images of cells after the addition of 10 mU/mL of Chondroitinase ABC. **C)** Confocal images of cells after the addition of 20 mU/mL of Chondroitinase ABC. **D)** Confocal images of cells after the addition of 30 mU/mL of Chondroitinase ABC. **E)** Positive control of cells with Heparan Sulphate. **F)** Confocal images of cells after the addition of 2 U/mL of Heparinase I and III. For the preceding assays, 30 mU/mL of Chondroitinase ABC and 2U/mL of Heparinase I and III were selected. Imaging was performed using a MolecularDevices ImageXpress Micro high-content imaging system, and image analysis was carried out using Fiji software (version 1.51) (n = 3). Scale bar represents 103 μm.

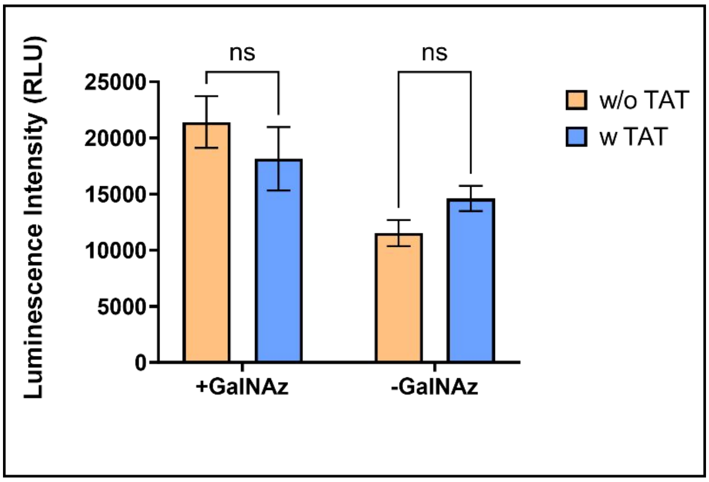

Figure S6: Determination of TAT endocytosis at 4℃ using DHL assay. *Statistical analysis was performed by two-way ANOVA. (ns) not significant: p>0.05. Data is expressed as mean ± SD (n = 3).*

2. Materials and Experimental Methods

2.1 Materials

DBCO-CA (CAS-no. 1808119-16-5) was purchased from Iris Biotech GmBH. Halo-HiBiT was obtained from Promega (N3010). Anti-Halo-HiBiT antibody was bought from Promega (N7200). Mouse IgM secondary antibody was purchased from Thermo Fischer (62-6520). Nano-Glo® Halo-HiBiT Extracellular Detection System was bought from Promega. 4–20% Mini-PROTEAN® TGX Stain-Free™ Protein Gels, 10 well, 30 µL #4568093 were obtained from BIO-RAD. Trans-Blot Turbo Midi 0.2 µm Nitrocellulose Transfer Packs were purchased from BIO-RAD. Rhodamine X 6-azide (CAS no. 1422178-12-8) was purchased from Lumiprobe. N-Azidoacetylgalactosamine-tetraacylated (GalNaz) (CAS no. 653600-56-7) was obtained from Sigma Aldrich. CellTiter-Glo® Luminescent Cell Viability Assay was purchased from Promega. DBCO-Cy5 was bought from Sigma-Aldrich (777374). Chondroitinase ABC from Proteus vulgaris was obtained from Sigma Aldrich. Heparinases I and III Blend from Flavobacterium heparinum was obtained from Sigma-Aldrich. Chondroitin Sulfate Monoclonal Antibody (CS-56) was purchased from Thermo Fischer (MA1-83055). N-sulfated Heparan Sulfate Monoclonal Antibody (HepSS-1), Biotin was purchased from Thermo Fischer (MA5-46186). Bovine Serum Albumin (BSA) was purchased from Sigma-Aldrich. 4% paraformaldehyde was purchased from Thermo Fischer. 4′,6-Diamidine-2′-phenylindole dihydrochloride (DAPI) was bought from Sigma Aldrich. The HIV-1 Tat Peptide was obtained from Sigma-Aldrich. EDTA 0.5 M pH 8.0 was bought from Thermo Fischer. Phosphate Buffer Saline (PBS 10x [-]Ca^{2+}[-]Mg^{2+}) was obtained from Gibco (SH30258.01). All other reagents were of analytical or chromatography grade.

2.2 Synthesis and Evaluation of DBCO-HiBiT Conjugation

2.2.1 ESI-Q-TOF-LC-MS/MS

To determine the conjugation efficiency of DBCO-CA to Halo-HiBiT, triplicates of DBCO-HiBiT or Halo-HiBiT were made. For the DBCO-HiBiT sample, 5 nM of DBCO-CA was mixed with 5 nM of Halo-HiBiT (1:1 ratio) in the desired volume of Hanks' Balanced Salt Solution (HBSS) buffer (SH30588.01, Gibco) with Ca^{2+} and Mg^{2+} and incubated for 1 h. For the Halo-HiBiT sample, 5 nM of Halo-HiBiT was incubated in HBSS buffer with Ca^{2+} and Mg^{2+} for 1 h. After which, ESI-Q-TOF-LC-MS/MS was performed using the following conditions:

Nine microliters (9 µL) of protein sample were injected into LCMS. Analytes were separated

using an Eksigent Ekspert 400 with a reverse C4 column (0.3 x 20 mm, Phenomenex, USA) in micro-level mode. The injected sample analytes were separated with a linear gradient of 10 min comprising an initial 1 min wash 3% solution B (0.1 % formic acid/acetonitrile), followed by a 5 min gradient from 3% to 95% of, and a 1 min stay at 95% B. The solution A was 0.1% formic acid in water, and the flow rate was set to 5 µL per min. The mass spectrometer was operated in TOF-MS mode (TripleTOF 6600 (Sciex, USA)) with the following settings: ion spray voltage at 5500 volts, ion source and curtain gases at 10 and 30 L/min, respectively. TOF-MS was acquired from m/z 400 to 2500. Following LC-MS acquisition, raw files were analyzed, and protein TIC (total ion current) chromatograms were deconvolved using PeakView software, version 2.2 (Sciex). Each sample injection was followed by at least one empty run to minimize the carryover.

2.2.2 Western Blot

3.6 Pg (within the detection range of the Anti-Halo-HiBiT antibody) of Halo-HiBiT and DBCO-HiBiT (ratio 1:1) complex were prepared in HBSS buffer with Ca^{2+} and Mg^{2+}. Next, the proteins were denatured and separated on a 4-12% SDS-polyacrylamide gel, followed by transfer onto a nitrocellulose membrane. Membranes were blocked for 1 h using EveryBlot Blocking Buffer (Bio-Rad) and incubated overnight at 4 °C with AntiHalo-HiBiT monoclonal antibody diluted 1:1000 (N7200; Promega). The next day, membranes were washed three times with 1× TBST and then incubated with mouse IgM secondary antibody (1:5000) for 1 h at room temperature. Protein bands were visualized using enhanced chemiluminescence (ECL) and imaged using auto exposure setting with a ChemiDoc XRS system (Bio-Rad, Hercules, CA, USA). The uncropped image of the blot can be referred to in section 2.10, figure S12.

2.2.3 Calibration Curve

To assess the retention of Halo-HiBiT function in the DBCO-HiBiT complex, a calibration curve was generated. To form the DBCO-HiBiT complex, Halo-HiBiT was incubated with DBCO-CA in a ratio of 1:1 in HBSS with Ca^{2+} and Mg^{2+}. A serial dilution (10^{-16}-10^{-13} mol) of fresh Halo-HiBiT (reference sample), Halo-HiBiT in HBSS with $Ca^{2+}$and Mg^{2+} (unbound sample), and DBCO-HiBiT complex (test sample) in HBSS with Ca2+ and Mg^{2+} was prepared, where the unbound and test samples were incubated at 37 °C for 1 or 24 h. The luminescence intensities were obtained using the Nano-Glo® Halo-HiBiT Extracellular Detection System according to the manufacturer's instructions and read on a Varioskan™ LUX multimode microplate reader. Briefly, LgBiT protein was diluted 1:100 in Nano-Glo®

Halo-HiBiT Extracellular Buffer, and Nano-Glo® Halo-HiBiT Extracellular Substrate was diluted 1:50 into the desired volume (depending on the number of samples) of Nano-Glo® Halo-HiBiT Extracellular Buffer at room temperature. The LgBiT protein and substrate mixture were added to each sample in a 1:1 ratio. Halo-HiBiT function retention was evaluated by comparing the luminescence intensity of the reference and unbound sample to the test sample across all used concentrations. All samples were made in triplicate.

2.2.4 Atomistic Molecular Dynamics Simulations

The starting structure of the Halo-HiBiT fusion protein was generated using the AlphaFold [1]. Dibenzocyclooctyne (DBCO-CA) was parameterized with the CHARMM-GUI Ligand & PDB Reader [2] [3]to ensure compatibility with the CHARMM36m force field [4] that was used to model Halo-HiBiT fusion protein. DBCO-CA was covalently linked to Halo aspartic acid located in the active site. The protein–ligand complex was embedded in a periodic cubic water box using the TIP3P water model [5]. Counterions were added to neutralize the system. All-atom molecular dynamics simulations were performed with the GROMACS simulation package [6]. After steepest-descent energy minimization and gradual equilibration, production simulations were carried out for up to 1 µs. Three replicates were run. Simulations were run in the NPT ensemble at a constant temperature of 298 K using the velocity-rescale thermostat [7] and a constant isotropic pressure of 1 bar maintained with the Parrinello–Rahman barostat [8]. Long-range electrostatics were treated with the Particle Mesh Ewald (PME) method [9] [10] with a real-space cutoff of 1.2 nm. Lennard-Jones (LJ) interactions were computed with a cutoff of 1.2 nm, and a force-switch function was applied between 1.0–1.2 nm to smoothly truncate van der Waals interactions. All hydrogen bonds were constrained with the LINCS algorithm [11]. A leapfrog integrator with a timestep of 2 fs was employed, and periodic boundary conditions were applied in all directions.

2.3 Evaluation of DBCO-CA accessibility using proximity-based BRET

The assay was performed in a 96-well plate. Briefly, Rhodamine X 6-azide (6-ROX, Lumiprobe) was titrated to different starting concentrations (0.03 µM-30 µM) in HBSS with $Ca^{2+}$and Mg^{2+}. DBCO-CA was mixed with a fixed Halo-HiBiT starting concentration of 15 nM in the desired initial concentrations (0.075 – 1500 nM for concentration-dependent analysis) and incubated for 1 h at 37°C in HBSS with $Ca^{2+}$and Mg^{2+}. Both solutions were added sequentially to the assay plate in a volume of 50 µL in the order of ROX 6-azide

followed by DBCO-HiBiT complex. To account for background signal, a no ligand control (with 0% fractional occupancy), comprising only Halo-HiBiT, was included and used as the baseline for BRET ratio calculation. To validate the assay, a Halo-HiBiT control (no alykne control), containing both Halo-HiBiT and ROX 6-azide, was used for comparative analysis. After incubating in the desired time intervals (0 - 60 minutes for time-dependent analysis or 60 minutes for concentration-dependent analysis), a mixture of LgBiT and Nano-Glo Substrate from the Nano-Glo® Halo-HiBiT Extracellular Detection System was diluted for a final volume of 50 µL and added according to the manufacturer's instructions and read on Varioskan™ LUX multimode microplate reader. The final dilution of all controls was 1:3.

BRET spectrum was realized by comparing the luminescence signal of the no alkyne control with 6-ROX, DBCO-HiBiT with 6-ROX, and Halo-HiBiT without 6-ROX along 370-700 nm wavelengths.

BRET ratios were calculated by dividing the acceptor emission at 591 nm by the donor emission at 450 nm for each sample. To correct for background, values obtained from the no-ROX control were subtracted from each corresponding sample. The resulting BRET ratios were then converted to milliBRET units (mBRET) by multiplying by 1000. The following equation illustrates this:

$$mBRET = \left(\left(\frac{A_{sample}}{D_{sample}}\right) - \left(\frac{A_{no-ROX}}{D_{no-ROX}}\right)\right) \times 1000$$

Where:

- A = Acceptor emission (591 nm)
- D = Donor emission (450 nm)

2.4 Optimization of GalNaz Metabolic Labeling

2.4.1 Cell culture

Mouse mammary carcinoma 4T1 cells (Mus musculus, ATCC catalogue no. CRL-2539, American Type Culture Collection; RRID: CVCL_0125) were cultured in RPMI-1640 medium (Gibco, SH30027.01) supplemented with 10% heat-inactivated fetal bovine serum (FBS) (Gibco, 17479633), 1% penicillin (100 IU mL^{-1}) and streptomycin (100 µg mL^{-1}) (Gibco, 15140122), and 1% non-essential amino acids (NEAA) (Gibco, SH30238.01). Cells

were maintained at 37 °C in a 95% humidified incubator with 5% CO_2. Cells were used in passage number between 20 and 35.

2.4.2 Viability Assay

In vitro cell viability assays were used to evaluate GalNaz safety using CellTiter-Glo® luminescent cell viability assay. Cells (4T1 or CHO) were seeded in a 96-well plate at a density of 1 × 10^4 cells per well in cell culture medium. Subsequently, GalNaz was added at different concentrations (50 – 500 μM) and incubated for 48 h. The produced luminescence intensity is directly proportional to the amount of ATP produced by viable cells. Varioskan™ LUX multimode microplate reader was used to measure such intensities according to the manufacturer's instructions (n = 3)

2.4.3 DBCO-Cy5 labeling and flow cytometry analysis

To label azides on cell membranes, a concentration and time-dependent evaluation was performed. Briefly, a seeding density of 5 × 10^4 cells per well was placed in a 24-well plate in 500 μL of culture media and allowed to grow for 24 h. After which, the culture media was removed, and cells were incubated with 100 μM GalNaz for 48 h. This was followed by a further removal of media and incubation with DBCO-Cy5 in different concentrations (5-500 nM) or different time intervals (0.1-4 h) at 500 nM concentration in culture media. To allow for background signal comparison, a non-metabolically labeled control (without GalNaz) was used. To obtain data on metabolic labeling, flow cytometry was employed. Briefly, cells were washed twice with 1x PBS-EDTA, then trypsinized. Pellet collection was done via centrifugation at 200 ×g for 5 min, followed by redispersion in 5% FBS in 1x PBS. Measurement was performed using a BD LSRFortessa™ flow cytometer (n = 3).

2.4.4 DHL assay labeling analysis

DHL luminescent assay was evaluated and optimized in vitro in cell studies through concentration and time-dependent assays. Following metabolic labeling in a 96-well plate format, 5 nM DBCO-CA was incubated with 5 nM Halo-HiBiT in the desired volume of HBSS buffer with Ca^{2+} and Mg^{2+} for 1 h to allow conjugation. Subsequently, the culture media was removed, and the cells were washed once with 1x HBSS Buffer with $Ca^{2+}$and Mg^{2+}. and replaced by 90 μL of OptiMem without phenol red but with 1% FBS (31985062, ThermoFischer). 10 μL of DBCO-HiBiT was added to each well, giving the desired final concentrations (5 – 500 nM) for the concentration-dependent study, and incubated for different time intervals (0 – 24 h) at 37 °C in 5% CO2 for the time-dependent study. After

which, cells were washed twice with HBSS with $Ca^{2+}$and Mg^{2+} and incubated in OptiMem for 1 h at 37 °C 5% CO_2. Finally, a mixture of LgBiT and Nano-Glo Substrate was added according to the manufacturer's instructions and read on a Varioskan™ LUX multimode microplate reader.

2.4.5 Evaluation of membrane non-specific binding using Halo-GFP

To mimic the upcoming DBCO-HiBiT assay, the luminescent platform was replaced by a fluorescent format by replacing Halo-HiBiT with a fluorescent Halo-GFP fusion protein. The modified HaloTag was fused to the N-terminus of GFP, serving as a model for a generic bioactive ligand. Following cell membrane metabolic labeling in a 24-well plate format, DBCO-CA was allowed to react with Halo-GFP in a 1:1 ratio at a concentration of 500 nM for 1 h in HBSS with Ca^{2+} and Mg^{2+}, followed by media removal and incubation with DBCO-CA-GFP complex for 1 h. A non-metabolically labeled control was used to compare the background. Measurements were read using flow cytometry under the previously mentioned conditions in section 2.3.3.

2.4.6 Coarse-Grained Molecular Dynamics Simulations

Coarse-grained molecular dynamics simulations were conducted to investigate interactions between Halo-HiBiT and POPC or POPC+GM3 lipid bilayers as simplified models of the plasma membrane. Initial bilayer structures, each containing approximately 200 lipids per leaflet, were generated using the CHARMM-GUI interface [3] [2]The Halo-HiBiT protein was subsequently placed in the aqueous phase above the membranes to study protein–membrane interactions.

Simulations employed the MARTINI 3 force field with standard parameters, including a Lennard-Jones cut-off of 1.1 nm and a dielectric constant of 15 [12]. Each system (POPC and POPC+GM3, ratio 10:1) was simulated in two independent replicates to ensure reproducibility. Systems were energy-minimized with the steepest descent methods prior to production runs. Temperature was maintained at 298 K using the velocity-rescaling thermostat [7], and semi-isotropic pressure coupling at 1 bar was applied using the Parrinello-Rahman barostat [8]. Protein, lipids, and water were all separately coupled to the heat path. Time step of 20 fs was used throughout [13].

Production simulations were carried out for up to 18 microseconds per replicate, allowing sufficient sampling of membrane dynamics and protein interactions. Trajectories were analyzed for Halo and Halo-HiBiT binding.

2.5 Optimization and validation of DHL assay

2.5.1 GalNaz labeling specificity evaluation

To study the specificity of GalNaz labeling of glycosaminoglycans (GAGs), chondroitin sulfate or heparan sulfate was removed using glycosidases (Chondroitinase ABC or Heparinase I and III), and the luminescent signal was assayed in their absence. To this end, inhibition of the expression of these glycans was assessed by immunostaining. Briefly, 4T1 cells were seeded in a 96-well black phenoplate (6055300; Revvity) at a density of 1 × 10^4 cells per well. After incubation for 24 h at 37 °C 5% CO 2, the media was removed, and cells were washed once with HBSS with Ca^{2+} and Mg^{2+}. Before the addition of chondroitinase ABC, its final dilutions were made in 50 mM Trizma® HCl, pH 8.0, with 60 mM sodium acetate and 0.02% BSA in serum-free, antibiotic-free media freshly just before use according to the manufacturer's instructions. After which, the cells were incubated for 3 h with chondroitinase ABC in different concentrations (10 - 30 mU ml−1; Sigma-Aldrich) or heparinase I and III (2 U ml−1 corresponding to 0.0033 IU ml−1; Sigma-Aldrich) in serum-free medium. Then, the cells were washed once with serum-free media and fixed with 4% paraformaldehyde for 15 min at room temperature. Following fixation, the cells were washed twice with HBSS buffer with Ca2+ and Mg2+, and blocked with 3% BSA blocking buffer for 1 h. Immunostaining was done by removing the blocking buffer and incubating with a primary antibody against heparan sulphate or a primary antibody against chondroitin sulphate with a dilution of 1:100 in 3% BSA overnight at 4 ℃. After which, cells were washed twice with blocking buffer and incubated with FITC-labeled anti-mouse IgM antibody with a 1:100 dilution for 1 h. Finally, cells were washed with HBSS with $Ca^{2+}$and Mg^{2+} once and were stained with 1 µg/ml 4′,6-diamidino-2-phenylindole (DAPI) for 3 min, which was then replaced by HBSS buffer with $Ca^{2+}$and Mg^{2+} for imaging purposes. Three replicates were made per sample. Imaging was performed using a MolecularDevices Image Xpress Micro high-content imaging system, and image analysis was carried out using Fiji software (version 1.51).

After determining the effective concentrations to remove these GAGs, each glycosidase (chondroitinase ABC or Heparinases I and III) was incubated with cells in a 96-well plate for

a DHL assay or in a 24-well plate for DBCO-Cy5 flow cytometry for 3 h. After which, cells were washed once with HBSS with $Ca^{2+}$and Mg^{2+}, and the DHL assay or DBCO-Cy5 flow cytometry protocol was performed as described previously.

2.5.2 Evaluation and validation of DHL assay ability to determine endocytosis

To evaluate and validate the ability of the assay to detect glycoprotein-mediated endocytosis in both 4T1 cells, CHO-K1, and pgsB618, a model peptide, TAT (H2N-YGRKKRRQRRR-$CONH_2$), was used. Briefly, following metabolic labeling, the media was replaced by 90 µL OptiMem, and 10 µL of DBCO-HiBiT complex was added for a final concentration of 5 nM, incubated for 1 h at 37 °C, 5% CO 2. The cells were washed twice with HBSS with Ca^{2+} and Mg^{2+}. TAT was added in OptiMEM for a final concentration of 10 µM per well. After 1 h incubation with peptide either at 37°C or 4°C, the media was removed, and the cells were washed twice with HBSS with $Ca^{2+}$and Mg^{2+}. Change in signal was assayed using Nano-Glo® Halo-HiBiT Extracellular Detection System according to the manufacturer's instructions and read on Varioskan™ LUX multimode microplate reader. Endocytosis was expected to be directly correlated with the decrease in signal. To account for the background signal, a non-metabolically labeled control (without GalNaz) was included. To assay endocytosis-dependent uptake, a metabolically labeled control lacking the TAT peptide was used. Additionally, to evaluate non-specific interaction between LgBit and the peptide, a metabolically labeled control (with and without TAT) was treated with LgBit alone, without the DBCO-HiBiT conjugate.

2.6 Determining glycan-CPP interactions and immunostaining

2.6.1 Validation of glycan-mediated endocytosis using CPPs

The DHL assay was used to relatively quantify CPP-glycan interactions. For this, 4 CPPs were used: TAT (H2N-YGRKKRRQRRR-CONH2), P1 (H2N-RQIKIWFQNRRMKWKK-CONH2), (R/W9) (RWWRRWRR), and R9 (RRRRRRRRR). The change in signal after peptide addition was assayed on 4T1 cells as discussed previously in section 2.5.3.

Moreover, chondroitin sulfate or heparan sulfate was removed to assay quantification in their absence as previously mentioned. After which, the DHL assay was performed to determine the relative signal change for each CPP after removal of the mentioned GAGs, as previously described.

2.7 Data analysis methods

2.7.1 Statistical analysis

Data are presented as the mean ± standard deviation (SD) from at least three independent experiments. Statistical analyses were performed using GraphPad Prism (version 10.1.2, CA, USA). Depending on the dataset, one-way or two-way ANOVA followed by Tukey's post-hoc test, or an unpaired *t*-test, was used to assess statistical significance. Differences were considered significant at $p \leq 0.05$.

2.7.2 Dynamic simulation analysis

DBCO-CA's solvent accessible surface was analyzed by using gmx sasa program included in the GROMACS simulation package software. Changes in secondary structure content were monitored throughout the trajectory using DSSP [14]. Molecular visualization and inspection of trajectories were carried out using VMD [15]The number of contacts (the cutoff was 0.6 nm) between Halo and POPC or GM3 was calculated by utilizing the gmx mindist program included in the GROMACS package.

2.8 Cell-penetrating peptide synthesis

2.8.1 Reagents and solvents

Fmoc- and side chain-protected L-amino acids (Fmoc-Ala-OH, Fmoc-Arg(Pbf)-OH, FmocAsn(Trt)-OH, Fm-oc-Asp(O*t*Bu)-OH, Fmoc-Cys(Trt)-OH, Fmoc-Gln(Trt)-OH, FmocGlu-(O*t*Bu)OH, Fmoc-Gly-OH, Fmoc-His(Trt)-OH, Fmoc-Ile-OH, Fmoc-Leu-O-H, FmocLys-(Boc)-OH, Fmoc-Met-OH, Fmoc-Phe-OH, Fmoc-Pro-OH, Fmoc-Ser(*t*Bu)-OH, FmocTh-r(*t*Bu)-OH, FmocTrp(Boc)-OH, Fmoc--Tyr(*t*Bu)-OH, Fmoc-Val-OH) were purchased from Bachem AG; *O*-(7-azabenzotriazol-1-yl)-*N,N,N',N'*-tetramethyluronium hexafluorophosphate (HATU) was purchased from Bachem AG and (7-azabenzotriazol-1-yloxy)tripyrrolidinophosphonium hexafluorophosphate (PyAOP) were purchased from Advanced ChemTech CreoSalus; *N*,*N*diisopropylethylamine- ($i$$Pr_2$NEt, DIPEA, 99.5%) and acetic anhydride (Ac_2O, ≥98%) were purchased from Sigma-Aldrich; trifluoroacetic acid (TFA, for HPLC, ≥99.0%), triisopropylsilane (TIPS, 98%) and 3,6-dioxa-1,8-octane-dithiol (DODT, 95%) were purchased from Sigma-Aldrich. *N,N*-Dimethylformamide- (DMF) was purchased from VWR International (Avantor) and treated for >24 h with Aldramine trapping packets purchased from Chemassist Corportation.; dichloromethane (DCM, ≥99.8%) was

purchased from Fisher Scientific Ltd. Diethyl ether was purchased from Honeywell Riedel-de Haën. Acetonitrile (MeCN, for HPLC gradient grade, ≥99.9%) was purchased from Sigma-Aldrich. NovaPEG Rink Amide resin (0.41 mmol/g loading) was purchased from the Novabiochem-line from Sigma-Aldrich Canada Ltd.

2.8.2 Automated flow-based peptide synthesis (AFPS)

Peptides were synthesized on an automated-flow system built in the Hartrampf lab, which is similar to the published AFPS system [16]. Capitalized letters refer to L-amino acids. Unless otherwise noted, the following settings were used for peptide synthesis: flow rate = 20 mL/min for coupling and deprotection steps (as specified), wherein the reactor base (containing resin) was kept at 90 °C, with pre-activation at 90 °C or 60 °C (heating loop) as specified. The standard synthetic cycle involves a first step of prewashing the resin at 90 °C for 60 s at 20 mL/min. During the coupling step, three HPLC pumps are used: a 50 mL/min pump head pumps the activating agent, a second 50 mL/min pump head pumps the amino acid, and a 5.0 mL/min pump head pumps *i*Pr_2NEt (*neat*). The 50 mL/min pump head pumps delivered 0.398679 mL of liquid per pump stroke; the 5.0 mL/min pump head pumps 3.9239 × 10^{-2} mL of liquid per pump stroke.

All peptides were prepared by AFPS on the specified resin, and standard Fmoc/*t*Bu protected amino acids (0.40 M in DMF, 0.20 M final concentration) were coupled using HATU (0.38 M in DMF, 0.19 M final concentration) or PyAOP (0.38 M in DMF, 0.19 M final concentration) with DIPEA (delivered *neat*, approx. 0.27 M final concentration).

For amino acids D, E, F, G, I, K, L, a total volume of 6.4 mL of the "coupling solution" (i.e., amino acid [0.20 M], HATU or PyAOP [0.19 M], and DIPEA [0.27 M] in DMF) was applied for each coupling. For amino acids A, C, R, S, T, a total of 10.4 mL of "coupling solution" was applied for each coupling. Removal of the N^{α}-Fmoc group was achieved using 20% piperidine with 1% formic acid in DMF (6.4 mL, *v/v/v*) at a flow rate of 20 mL/min, with preheating at 90 °C for all Fmoc-protected amino acids except C, for which preheating of the deprotection solution was at 60 °C. Between each coupling and deprotection step, the resin was washed with DMF (32 mL) at a flow rate of 20 mL/min with preheating at 90 °C for all amino acids except C, for which the DMF was preheated at 60 °C. After completion of the peptide sequence, the resins were manually washed with DCM (3 × 5 mL) and dried under reduced pressure.

2.8.3 TFA-mediated peptidyl-resin cleavage and global deprotection

The peptides were cleaved using a solution of TFA/TIPS/DODT/H_2O (94:1:2.5:2.5, *v*/*v*/*v*/*v*, 1–3 mL) for 2 h at 23 °C with gentle shaking. The peptides were precipitated with ice-cold diethyl ether and isolated after centrifugation (2 × 15 mL). The resulting peptide pellets were briefly dried under a light stream of N_2, then dissolved in an aqueous solution containing 10–50% MeCN and 0.1% TFA, and lyophilized. Crude peptides were then analyzed by LC-HR-ESI-MS.

2.8.4 Liquid Chromatography with High-Resolution Electrospray Ionization Mass Spectrometry (LC-HR-ESI-MS)

For the determination of peptide masses by LC-MS, the filtered peptide solution was diluted in 10–50% acetonitrile (MeCN) in water with 0.1% TFA (60-500 µL) to a final concentration of approximately 0.1 mg/mL. The samples were analyzed on an Acquity UPLC (Waters, Milford, USA), which is connected to an Acquity eλ diode array detector and a Synapt G2 HR-ESI-QTOF-MS (Waters©, Milford, USA). Separation was carried out on an Acquity BEH C8 HPLC column (1.7 µm particle size, 2.0 × 50 mm, Waters©), which was at room temperature, with a sample injection volume of 5 µL. The elution was performed at a flow rate of 0.4 mL/min with solvent A: H_2O + 0.02% formic acid + 0.04% TFA and solvent B: MeCN + 0.04% formic acid + 0.02% TFA following the LC-MS

> **gradient:** isocratic at 3% Solvent B for 3 min, followed by a linear gradient of 3–95% Solvent B over 9 min, followed by isocratic at 95% Solvent B for 1 min.

Ion source parameters for ESI were: positive ionization mode, capillary voltage 3.0 kV, sampling cone 40 V, extraction cone 4 V, N_2 cone gas 4 L/h, N_2 desolvation gas 800 L/min and source temperature 120 °C. Parameters for the mass analyzer in resolution mode were: mass range 150–3000 *m/z* with a scan rate of 1 Hz; mass calibration to <2 ppm within 50–2500 *m/z* with a 5mM aq. soln. of HCO_2Na, lock masses: *m/z* 195.0882 (caffeine, 0.7 ng/mL) and 556.2771 (Leu-enkephalin, 2 ng/mL). All mass spectra show deconvoluted masses from the raw *m/z* values, calculated using Mestrelab Research S.L.© MestReNova v. 14.1 Mnova MS Suite. Purity based on LC-HR-ESI-MS was calculated by calculating the Area Under the Curve (AUC) of the desired product peak as a percentage of the AUC of all peaks (within 2–9 min) of the total absorbance chromatogram (λ = 214 nm). Monoisotopic and average masses for uncharged molecules were calculated using ChemDraw Version 18.2.

2.8.5 Semi-Preparative Reverse-Phase High Performance Liquid Chromatography (RP-HPLC)

Semi-preparative RP-HPLC was performed on a Shimadzu prominence HPLC system (Shimadzu Corp., Japan) with a CBM-40 system controller module, an FRC-10A fraction collector, two LC-20AR pumps, and an SPD-40 UV/VIS detector, using an Agilent Zorbax 300SB-C18 Semi-Preparative column (9.4 × 250 mm, 5 µm particle size) kept at 23 °C, with a flow rate of 3.5 mL/min. A binary solvent system was used, wherein Solvent A was H_2O containing 0.1% TFA, and Solvent B was MeCN containing 0.1% TFA. Purifications were executed using the following gradient:

> **Semi-preparative RP-HPLC gradient:** Isocratic at 5% Solvent B for 5 min, then linear gradient of 5–95% Solvent B over 60 min, followed by isocratic at 95% for 5 min.

2.8.6 Synthesis of H_2N-RQIKIWFQNRRMKWKK-$CONH_2$

The peptide H2N-RQIKIWFQNRRMKWKK-CONH2 was synthesized on commercially available Rink Amide NovaPEG resin (0.41 mmol/g, 121.3 mg, 50 µmol) using the standard AFPS protocol (Section 2.8.2) (Figure S6). Total synthesis time to afford the resin-bound peptide was approximately 0.8 h. Cleavage of the peptidyl-resin (100 mg, approx. 32 µmol) according to the cleavage protocol described in Section 2.8.3 afforded the crude peptide as a colorless solid (61.1 mg). The crude peptide was purified according to the protocol described

in Section 2.8.5 to yield the pure peptide as a colorless solid (5.2 mg, 7%, >99% purity, mass confirmed by LC-HR-ESI-MS (Figure S7).

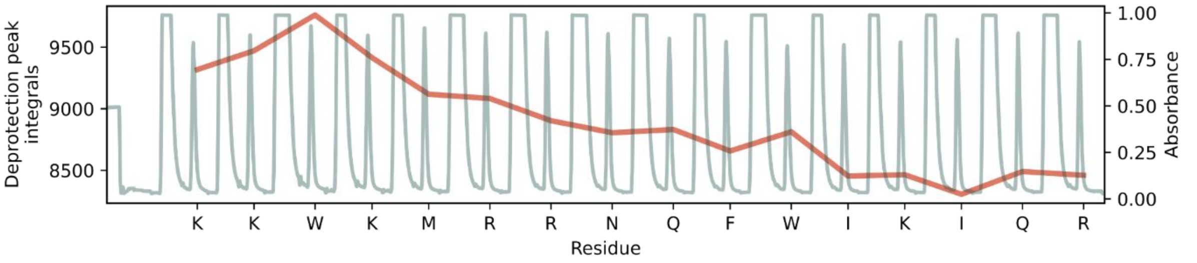

Figure S6. UV trace (λ = 310 nm) from AFPS **of H_2N-RQIKIWFQNRRMKWKK-$CONH_2$** (green) and deprotection peak integrals (red).

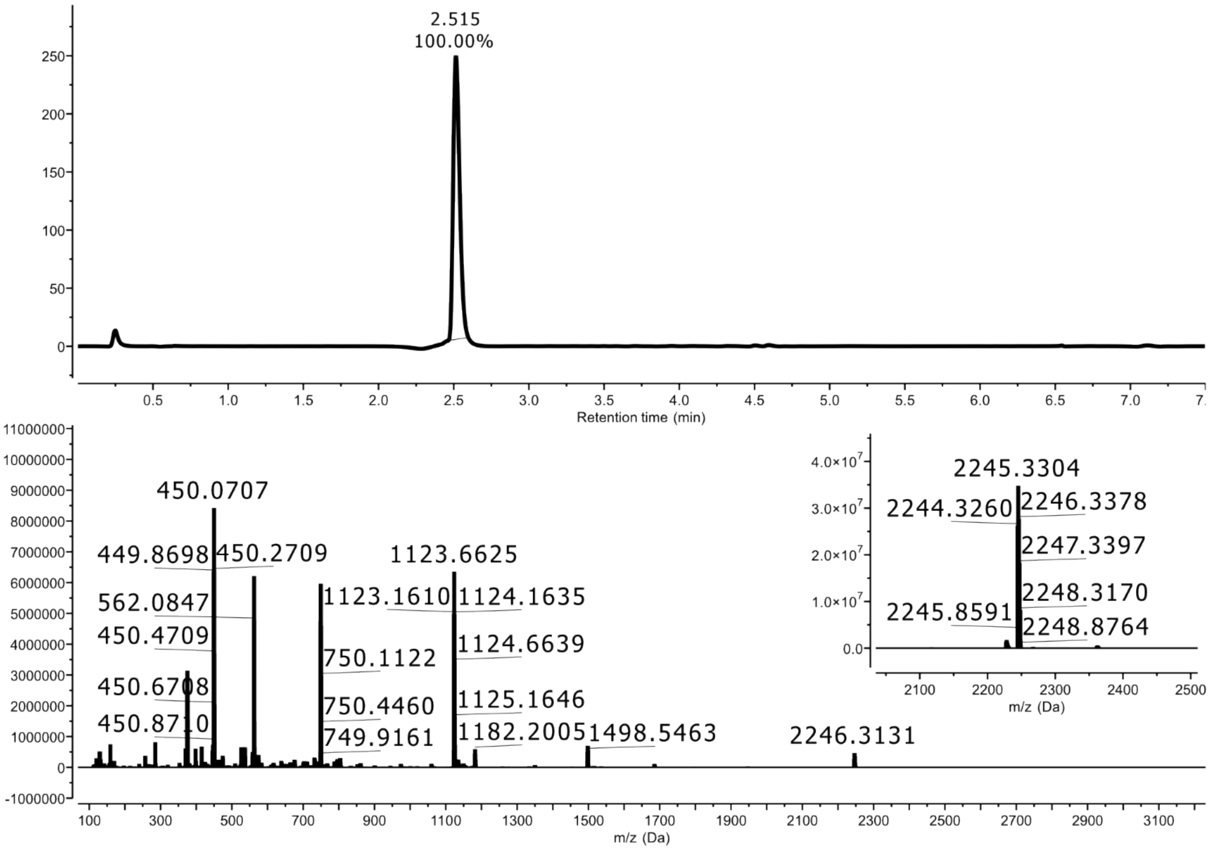

Figure S7. LC-HR-ESI-MS of purified H_2N-RQIKIWFQNRRMKWKK-$CONH_2$. Top: UV chromatogram (214 nm) of H_2N-RQIKIWFQNRRMKWKK-$CONH_2$; Rt 2.52 min. **Bottom:** ESI-MS spectrum found within Rt 2.52 min (insert: deconvoluted masses). Monoisotopic mass (ESI+) calcd. for $C_{104}H_{169}N_{35}O_{19}S$ 2244.3055, found 2244.3260. Measured according to protocol in **Section 2.8**.

2.8.7 Synthesis of H_2N-RRRRRRRRR-$CONH_2$

The peptide H_2N-RRRRRRRRR-$CONH_2$ was synthesized on commercially available Rink Amide NovaPEG resin (0.41 mmol/g, 152.2 mg, 62 µmol) using the standard AFPS protocol (**Section 2.8.2**) (**Figure S8**). Total synthesis time to afford the resin-bound peptide was approximately 0.5 h. Cleavage of the peptidyl-resin (150 mg, approx. 36 µmol) according to the cleavage protocol described in **Section 2.8.3** afforded the crude peptide as a colorless solid (43 mg). One portion (20 mg) of the crude peptide was purified according to the protocol described in **Section 2.8.5** to yield the pure peptide as a colorless solid (2.2 mg, 9%, >95% purity, mass confirmed by LC-HR-ESI-MS (**Figure S9**).

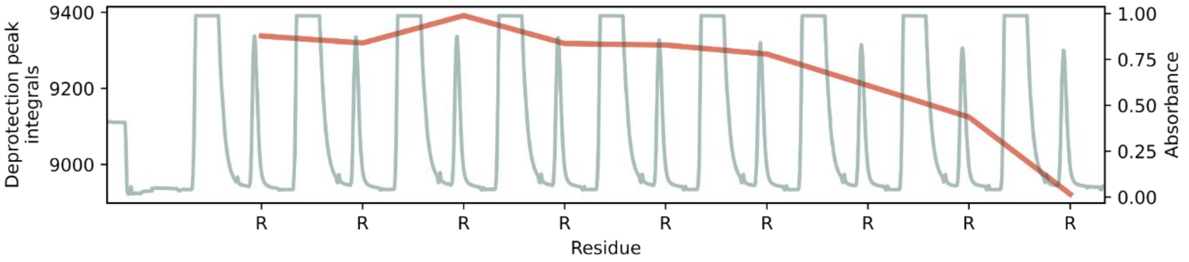

Figure S8. UV trace (λ = 310 nm) from AFPS of **H2N-RWWRRWRR-CONH2** (green) and deprotection peak integrals (red).

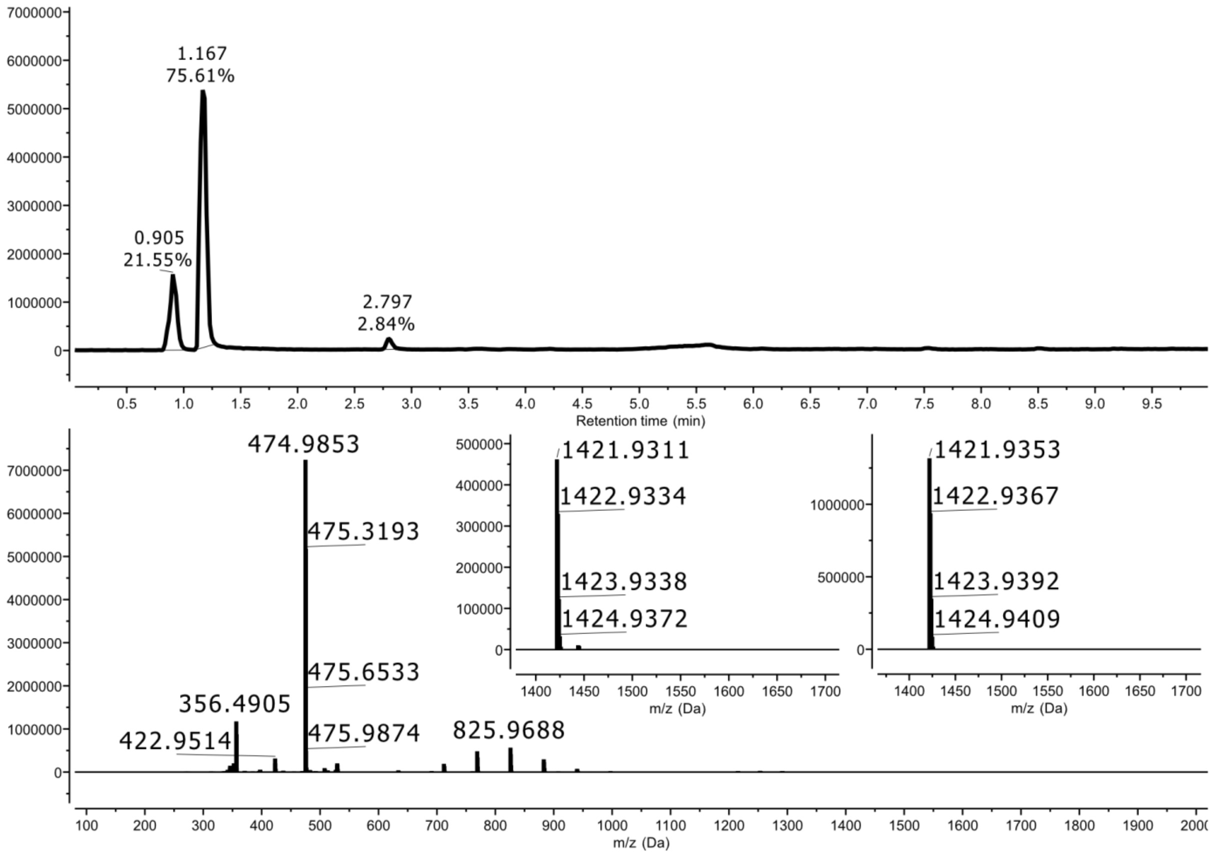

Figure S9. LC-HR-ESI-MS Profile of purified H_2N-RRRRRRRRR-$CONH_2$. Top: TIC chromatogram of H_2N-RRRRRRRRR-$CONH_2$; Rt 0.91 & 1.17 min. **Bottom:** ESI-MS spectrum found within Rt 0.60 – 1.50 min (insert left: deconvoluted masses of peak at Rt 0.91 min, insert right: deconvoluted masses of peak at Rt 1.17 min). Monoisotopic mass (ESI+) calcd. for $C_{54}H_{111}N_{37}O_9$ 1421.9365, found 1421.9311 (insert left) & 1421.9353 (insert right).

2.8.8 Synthesis of H_2N-RWWRRWRR-$CONH_2$

The peptide H_2N-RWWRRWRR-$CONH_2$ was synthesized on commercially available Rink Amide NovaPEG resin (0.41 mmol/g, 153.3 mg, 63 µmol) using the standard AFPS protocol (**Section 2.8.2**) (**Figure S10**). Total synthesis time to afford the resin-bound peptide was approximately 0.4 h. Cleavage of the peptidyl-resin (150 mg, approx. 38 µmol) according to the modified cleavage protocol described in **Section 2.8.3** (no addition of TIPS, instead 3.5% water) afforded the crude peptide as a colorless solid (47 mg). One portion (20 mg) of the crude peptide was purified according to the protocol described in **Section 2.8.5** to yield the pure peptide as a colorless solid (5.1 mg, 20%, >95% purity, mass confirmed by LC-HR-ESI-MS (**Figure S11**).

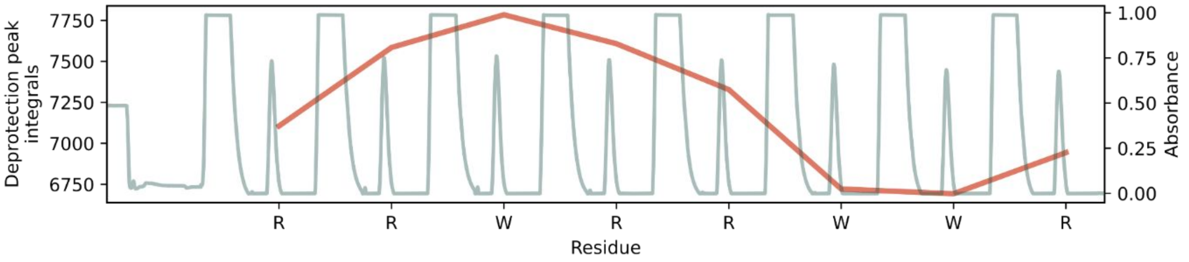

Figure 3UV trace (λ = 310 nm) from AFPS of **H2N-RWWRRWRR-CONH2** (green) and deprotection peak integrals (red).

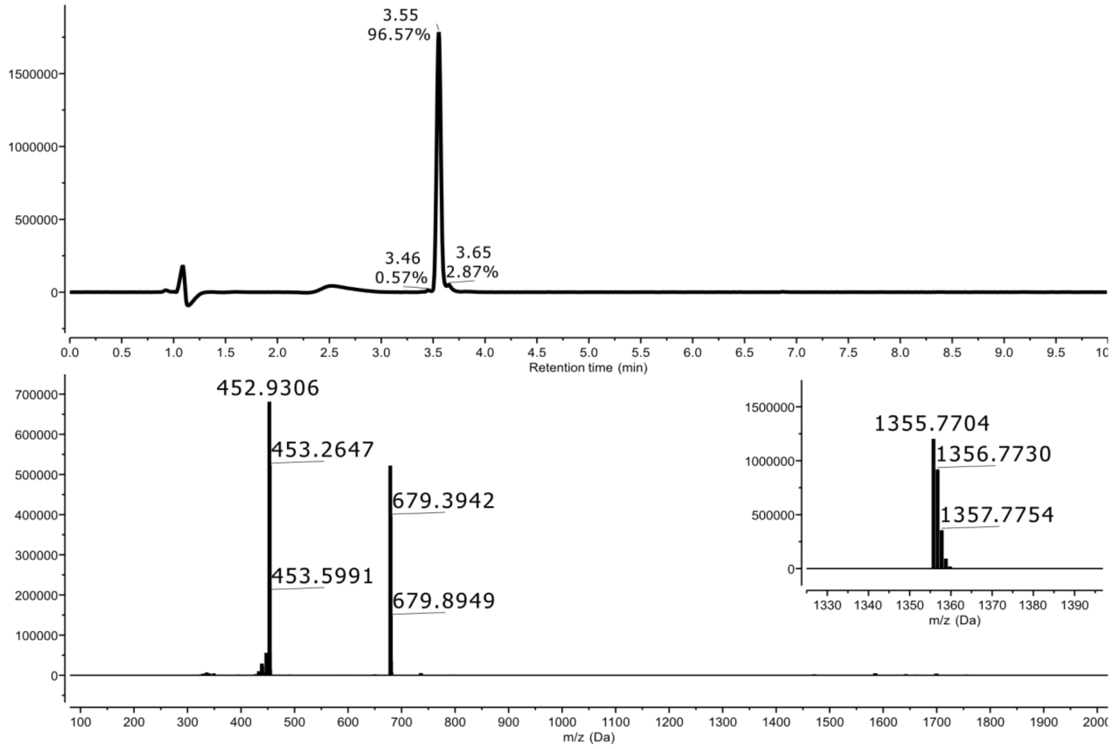

Figure S11. LC-HR-ESI-MS Profile of purified H_2N-RWWRRWRR-$CONH_2$. Top: UV chromatogram (214 nm) of H_2N-RWWRRWRR-$CONH_2$; Rt 3.55 min. **Bottom:** ESI-MS spectrum found within Rt 3.55 min (insert: deconvoluted masses). Monoisotopic mass (ESI+) calcd. for $C_{63}H_{93}N_{27}O_8$ 1355.7700, found 1355.7704.

2.9 Recombinant Halo-GFP production and purification

Halo-GFP was produced in Escherichia coli using a stirred-tank bioreactor according to a previously established protocol (ref. [previous work]). Briefly, plasmid-transformed E. coli cells were first grown in 200 mL of FRS01 medium (10 g L^{-1} tryptone, 5 g L^{-1} yeast extract, 0.5 g L^{-1} $MgSO_4 \cdot 7H_2O$, 5 g L^{-1} glycerol, and 100 µg mL^{-1} ampicillin) at 30 °C for 24 h in a 1 L Erlenmeyer flask under shaking (120 rpm). The seed culture was used to inoculate a 2 L stirred-tank bioreactor (Biostat A Plus, Sartorius) to obtain an initial optical density at 600 nm (OD_{600}) of 0.1. The same medium composition was employed, except that the glycerol concentration was increased to 20 g L^{-1} to promote biomass accumulation. The culture was grown until $OD_{600} \approx 1$, then protein expression was induced by adding lactose (2 g L^{-1}). Bioreactor parameters were maintained as follows: temperature initially set at 30 °C and reduced to 20 °C after induction; dissolved oxygen tension (DOT) controlled at 25% air saturation via cascade regulation of agitation speed (120–1000 rpm) at a constant airflow rate of 2 L min^{-1} (1 vvm); pH was left to naturally decrease from 7.0 to 5.5 during cultivation. After 24 h of induction, cells were harvested by centrifugation (5500 ×g, 10 min, 4 °C) and resuspended in PBS (pH 7.3) supplemented with DNase I (10 µg mL^{-1}), PMSF (0.19 mg mL^{-1}), protease inhibitor, and EDTA, and lysed by sonication on ice (15 cycles × 30 s, 12% amplitude). Cell debris was removed by centrifugation (20,000 ×g, 30 min, 4 °C). The soluble fraction was subjected to purification under native conditions using glutathione affinity chromatography (Pierce Glutathione Superflow Agarose, Thermo Scientific), exploiting the GST fusion tag. After binding and washing, Halo-GFP was released by on-column cleavage with PreScission Protease (GE Healthcare) in cleavage buffer (50 mM Tris-HCl, 150 mM NaCl, 1 mM EDTA, 1 mM DTT, pH 8.0) for 16 hat 4 °C under gentle agitation. The cleaved HaloGFP was collected by washing the resin with 20 column volumes (CV) of cleavage buffer, and purity was verified by SDS-PAGE analysis and western blot.

2.10 Figure S12: Uncropped Blot for figure 1 in the main text

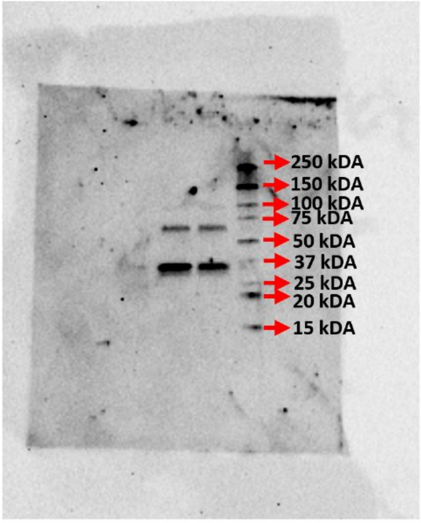

Figure S12: Uncropped Image of Western Blot for Halo-HiBiT and DBCO-HiBiT bands.

References

[1] J. Jumper, R. Evans, A. Pritzel, T. Green, M. Figurnov, O. Ronneberger, K. Tunyasuvunakool, R. Bates, A. Žídek, A. Potapenko, A. Bridgland, C. Meyer, S. A. A. Kohl, A. J. Ballard, A. Cowie, B. Romera-Paredes, S. Nikolov, R. Jain, J. Adler, T. Back, S. Petersen, D. Reiman, E. Clancy, M. Zielinski, M. Steinegger, M. Pacholska, T. Berghammer, S. Bodenstein, D. Silver, O. Vinyals, A. W. Senior, K. Kavukcuoglu, P. Kohli, D. Hassabis, Highly accurate protein structure prediction with AlphaFold.*Nature* **2021**, *596*, 583-589.

[2] J. Lee, X. Cheng, J. M. Swails, M. S. Yeom, P. K. Eastman, J. A. Lemkul, S. Wei, J. Buckner, J. C. Jeong, Y. Qi, S. Jo, V. S. Pande, D. A. Case, C. L. Brooks, III, A. D. MacKerell, Jr., J. B. Klauda, W. Im, CHARMM-GUI Input Generator for NAMD, GROMACS, AMBER, OpenMM, and CHARMM/OpenMM Simulations Using the CHARMM36 Additive Force Field.*Journal of Chemical Theory and Computation* **2016**, *12*, 405-413.

[3] S. Jo, T. Kim, V. G. Iyer, W. Im, CHARMM-GUI: a web-based graphical user interface for CHARMM.*J Comput Chem* **2008**, *29*, 1859-1865.

[4] J. Huang, S. Rauscher, G. Nawrocki, T. Ran, M. Feig, B. L. de Groot, H. Grubmüller, A. D. MacKerell, CHARMM36m: an improved force field for folded and intrinsically disordered proteins.*Nature Methods* **2017**, *14*, 71-73.

[5] W. L. Jorgensen, J. Chandrasekhar, J. D. Madura, R. W. Impey, M. L. Klein, Comparison of simple potential functions for simulating liquid water.*The Journal of Chemical Physics* **1983**, *79*, 926-935.

[6] M. J. Abraham, T. Murtola, R. Schulz, S. Páll, J. C. Smith, B. Hess, E. Lindahl, GROMACS: High performance molecular simulations through multi-level parallelism from laptops to supercomputers.*SoftwareX* **2015**, *1-2*, 19-25.

[7] G. Bussi, D. Donadio, M. Parrinello, Canonical sampling through velocity rescaling.*The Journal of Chemical Physics* **2007**, *126*.

[8] M. Parrinello, A. Rahman, Polymorphic transitions in single crystals: A new molecular dynamics method.*Journal of Applied Physics* **1981**, *52*, 7182-7190.

[9] T. Darden, D. York, L. Pedersen, Particle mesh Ewald: An N·log(N) method for Ewald sums in large systems.*The Journal of Chemical Physics* **1993**, *98*, 10089-10092.

[10] U. Essmann, L. Perera, M. L. Berkowitz, T. Darden, H. Lee, L. G. Pedersen, A smooth particle mesh Ewald method.*The Journal of Chemical Physics* **1995**, *103*, 8577-8593.

[11] B. Hess, H. Bekker, H. J. C. Berendsen, J. G. E. M. Fraaije, LINCS: A linear constraint solver for molecular simulations.*Journal of Computational Chemistry* **1997**, *18*, 1463-1472.

[12] P. C. T. Souza, R. Alessandri, J. Barnoud, S. Thallmair, I. Faustino, F. Grünewald, I. Patmanidis, H. Abdizadeh, B. M. H. Bruininks, T. A. Wassenaar, P. C. Kroon, J. Melcr, V. Nieto, V. Corradi, H. M. Khan, J. Domański, M. Javanainen, H. Martinez-Seara, N. Reuter, R. B. Best, I. Vattulainen, L. Monticelli, X. Periole, D. P. Tieleman, A. H. de Vries, S. J. Marrink, Martini 3: a general purpose force field for coarse-grained molecular dynamics.*Nat Methods* **2021**, *18*, 382-388.

[13] D. H. de Jong, G. Singh, W. F. D. Bennett, C. Arnarez, T. A. Wassenaar, L. V. Schäfer, X. Periole, D. P. Tieleman, S. J. Marrink, Improved Parameters for the Martini Coarse-Grained Protein Force Field.*Journal of Chemical Theory and Computation* **2013**, *9*, 687-697.
[14] W. Kabsch, C. Sander, Dictionary of protein secondary structure: Pattern recognition of hydrogen-bonded and geometrical features.*Biopolymers* **1983**, *22*, 2577-2637.
[15] W. Humphrey, A. Dalke, K. Schulten, VMD: Visual molecular dynamics.*Journal of Molecular Graphics* **1996**, *14*, 33-38.
[16] N. Hartrampf, A. Saebi, M. Poskus, Z. P. Gates, A. J. Callahan, A. E. Cowfer, S. Hanna, S. Antilla, C. K. Schissel, A. J. Quartararo, X. Ye, A. J. Mijalis, M. D. Simon, A. Loas, S. Liu, C. Jessen, T. E. Nielsen, B. L. Pentelute, Synthesis of proteins by automated flow chemistry.*Science* **2020**, *368*, 980-987.
